# Secondary Structure of Influenza A Virus Genomic Segment 8 RNA Folded in a Cellular Environment

**DOI:** 10.3390/ijms23052452

**Published:** 2022-02-23

**Authors:** Barbara Szutkowska, Klaudia Wieczorek, Ryszard Kierzek, Pawel Zmora, Jake M. Peterson, Walter N. Moss, David H. Mathews, Elzbieta Kierzek

**Affiliations:** 1 Institute of Bioorganic Chemistry, Polish Academy of Sciences, Noskowskiego 12/14, 61-704 Poznan, Poland; barbaraszutkowska90@gmail.com (B.S.); kwieczorek@ibch.poznan.pl (K.W.); rkierzek@ibch.poznan.pl (R.K.); pzmora@ibch.poznan.pl (P.Z.); 2 Roy J. Carver Department of Biochemistry, Biophysics and Molecular Biology, Iowa State University, Ames, IA 50011, USA; jakemp@iastate.edu (J.M.P.); wmoss@iastate.edu (W.N.M.); 3 Department of Biochemistry & Biophysics and Center for RNA Biology, 601 Elmwood Avenue, Box 712, School of Medicine and Dentistry, University of Rochester, Rochester, NY 14642, USA; david_mathews@urmc.rochester.edu

**Keywords:** influenza A virus, IAV, RNA virus, RNA secondary structure, RNA chemical mapping

## Abstract

Influenza A virus (IAV) is a member of the single-stranded RNA (ssRNA) family of viruses. The most recent global pandemic caused by the SARS-CoV-2 virus has shown the major threat that RNA viruses can pose to humanity. In comparison, influenza has an even higher pandemic potential as a result of its high rate of mutations within its relatively short (<13 kbp) genome, as well as its capability to undergo genetic reassortment. In light of this threat, and the fact that RNA structure is connected to a broad range of known biological functions, deeper investigation of viral RNA (vRNA) structures is of high interest. Here, for the first time, we propose a secondary structure for segment 8 vRNA (vRNA8) of A/California/04/2009 (H1N1) formed in the presence of cellular and viral components. This structure shows similarities with prior in vitro experiments. Additionally, we determined the location of several well-defined, conserved structural motifs of vRNA8 within IAV strains with possible functionality. These RNA motifs appear to fold independently of regional nucleoprotein (NP)-binding affinity, but a low or uneven distribution of NP in each motif region is noted. This research also highlights several accessible sites for oligonucleotide tools and small molecules in vRNA8 in a cellular environment that might be a target for influenza A virus inhibition on the RNA level.

## 1. Introduction

The influenza viruses are classified as types A, B, C, and D (IAV, IBV, ICV, and IDV) and belong to the *Orthomyxoviridae* family of viruses [1]. As a consequence of its transmission potential from a broad range of species to humans, IAV is considered one of the most dangerous pathogens around the globe [2]. It is estimated that the virus infects nearly 1 billion people annually, resulting in 290–650 thousand influenza-related deaths [3]. Currently, the most popular anti-influenza strategy is prevention through vaccination [4]. Vaccines are developed annually and some of them contain strains of inactive influenza virus that are predicted as the most probable to occur each year [5,6]. This prediction may be incorrect, leading to seasonal outbreaks. As an example, the A/California/04/2009 (H1N1) pandemic strain wreaked havoc and very quickly affected 88 million people worldwide [7]. Moreover, it is considered very threatening because humans have a lack of immunity to it from previous viral infections [4,8]. With these challenges in mind, the World Health Organization (WHO) started the Global Influenza Programme (2019–2030), focusing on prevention, control, and preparation for future influenza outbreaks.

The influenza A genome is constantly changing, owing to its high-level mutational potential. The lack of a proofreading function in the viral RNA-dependent RNA polymerase (vRdRp) is a source of genetic drift that can lead to new arising IAV subtypes [9]. Another possible source is a genetic shift, a phenomenon of interchange segments that occurs when two different strains of IAV meet in one cell [9]. Because of this viral diversity, there is a need to develop a more universal drug approach rather than a strain-specific one. Such an approach could focus on targeting the secondary structure of vRNA, impairing influenza replication at the transcriptional/translational level rather than the protein itself [5,10,11]. The antiviral effectiveness of protein-targeting drugs is decreasing with time as many resistant strains have evolved. Such an example is the numerous amantadine [12,13,14] or amantadine-oseltamivir-resistant strains that occur worldwide [15]. An RNA-focused approach will make a deeper understanding of influenza necessary, especially concerning the virus in its natural environment. In this work, we focused on answering questions about how the vRNA structure folds in the presence of viral and cellular components.

RNA structures are connected with numerous functions; this is a fact that holds for the influenza virus [16]. It has been proposed that many events during the IAV life cycle are RNA structure-dependent, including splicing, nuclear export, translation, vRNA packaging, recognition of host immune systems, and the switch between transcription and replication [17,18,19]. One of the well-known structural motifs in IAV is the secondary structure of the vRdRp promoter formed between the 3′ and 5′ UTR regions (12–13 nt) of every segment [17]. Notably, the promoter is highly conserved across all influenza strains [17,20]. As of today, a few different secondary structures of the promoter have been proposed: *panhandle*, *fork*, *corkscrew*, and *hook* [17,20]. All of these proposed structures are supported by independent experimental studies [20,21,22,23,24,25,26,27,28]. The intra-segmental pairing between the 3′ end and 5′ end was shown by cryo-EM studies and confirmed within all vRNAs in virio by Dadonaite et al. [22,29]. The *panhandle* structure was shown to be important for the binding of RIG-I in cells [30]. Additionally, it is now obvious that the promoter goes through structural changes during the replication cycle [26].

The secondary structures of vRNA segments 5, 7, and 8 and their conservation across type A strains have been determined in vitro by our group [31,32,33]. The existence of particular secondary structure RNA motifs from our previous research has also been confirmed via independent research [20]. Furthermore, knowledge of RNA secondary structures has allowed for the design of effective inhibitors such as antisense oligonucleotides (ASO) [31,34,35]. Although the proposed global folding is based on in vitro mapping data and supported by bioinformatics structure-sequence analysis, knowledge of folding in the cellular environment is needed for full interpretation and new important motif discovery.

Although in vitro probing provides useful knowledge of RNA structures, it does not concern structural changes impaired by the interaction of the RNA with other biomolecules. Each influenza’s viral RNA along with the viral proteins form an independent ribonucleoprotein complex (vRNP); a high-resolution structure of vRNP was resolved very recently [36]. Every vRNP complex consists of many copies of nucleoprotein (NP) and vRdRp complexes. It was confirmed that vRNA is not strictly attached to the NP core; some regions are exposed outside the vRNP complex [37,38,39,40]. Some of these regions are responsible for intersegmental vRNA–vRNA interactions [25]. Examples of such interactions include packaging signals responsible for the appropriate packaging of a complete subset of vRNP into nascent virions [41]. It was confirmed that induced mutations altering the predicted RNA structures in low-NP bound regions impaired the genome packaging, resulting in viral attenuation [42]. During the replication processes in a cell, viral RNAs interact as well with cellular RNAs and cellular RNA-binding proteins (RBPs) [43]. Nevertheless, the influenza vRNA’s secondary structure in the cellular context remains unexamined.

In this research, the secondary structure of segment 8 vRNA (vRNA8) of A/California/04/2009 (H1N1) was determined in the presence of cellular and viral components. Segment 8 encodes at least two non-structural proteins that are engaged in different stages of the viral life cycle [44]. Non-structural protein 1 (NS1) plays its role during viral infection, acting as a regulation factor during the splicing of other viral mRNAs [45] and blocking the host’s antiviral responses on many levels [45,46,47]. Understandably, NS1 is expressed at its highest levels in the early stages of cellular infection [47]. Our previous in vitro research led to the determination of secondary structural elements in segment 8 vRNA IAV that are conserved across many strains [34]. This research was used for the design of ASOs that were tested in cells, exhibiting a high level of IAV inhibition [34]. Unexpectedly, several oligonucleotides targeting vRNA8 designed based on in vitro structure were found to be ineffective. This could be the result of RNA intramolecular interactions or alternate RNA folding in a cellular environment, or both, resulting in the inaccessibility of these regions in living cells. Herein, chemical mapping of vRNA8 in the cellular environment coupled with bioinformatics analysis was used to discover new structural features that might be potentially crucial for the viral replication cycle. Indeed, our study confirmed that the structure of vRNA8 in the biological context folds partially differently than in vitro. Nonetheless, we confirmed that some previously established for different IAV strains in vitro and in virio vRNA8 motifs exist in cell lysate. Moreover, we performed wide-scale vRNA8 structure conservation analysis. The knowledge provided in this study could be used to better interpret the role of vRNA8 in viral processes in cells and lead to the design of more accurate and sophisticated antiviral therapies targeting viral RNA.

## 2. Results

### 2.1. Chemical Mapping of vRNA8 in Cell Lysates

Chemical mapping uses reagents that can react with RNA within accessible regions that are either single-stranded or locally flexible, or both. The method, coupled with a reverse transcription reaction that stops one nucleotide before the modified nucleotide, gives information about single-stranded regions and base pairing within the RNA. In this study, two different chemical reagents were used to probe vRNA8 in cell lysates: DMS (dimethyl sulfate), which can react with adenosine and cytosine (N1 or N3 position, respectively), and 1M7, which is one of the SHAPE (2′-hydroxyl acylation analyzed by primer extension) reagents that react at the 2′OH position of all accessible nucleotides. Lysates from infected MDCK cells were obtained as described in the Materials and Methods section of this paper and checked for the presence of viral proteins by Western blot analysis (Supplementary S1 Text: Figure S1). The level of infection was additionally evaluated by vRNA concentration calculation via qPCR (Supplementary S1 Text: Figure S4). Next, chemical probing was performed as described in the Materials and Methods section. Additionally, before reverse transcription, the isolated RNA was checked for integrity on a non-denaturing agarose gel which verified an intact total RNA and single vRNA8 product (890 nt long).

**Figure 1:**
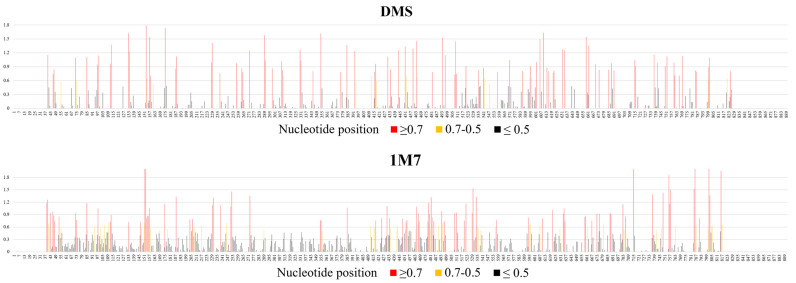
Average nucleotide reactivity distribution across vRNA8 A/California/04/2009. The vRNA8 chemical mapping experiments were performed in cell lysates at 37 °C with 1M7 (SHAPE) or DMS chemical reagents. The probing experiments were performed in three biological replicates of which a minimum of three technical replicates were made. High reactivities are marked with red (values ≥ 0.700), medium reactivities with yellow (values 0.500–0.700), and low reactivities (values ≤ 0.500) are marked with black. Regions of unknown reactivity due to the limitation of the read-out method were 1–38 nt and 818–890 nt (1M7); 1–38 nt and 828–890 nt (DMS).

Results of vRNA8 structure probing show that 89 nt were strongly modified with DMS (reactivities values ≥ 0.7) (20.48% of all A and C of vRNA8), 18 nt were moderately modified (reactivity values between 0.7–0.5), and 186 nt were weakly modified (reactivity values between 0.5–0.001). Mapping with 1M7 spanned 56% of all nucleotides. The results showed strong modification (reactivities ≥ 0.7) of 87 nt, medium modification (0.7–0.5) of 55 nt, and 357 nt that were weakly modified (Figure 1, Supplementary S1 Data: Table S2). The modifications were widely distributed across the whole segment, with regions of continuous strong modification adducts (defined here as the most reactive regions) and even more regions with no or very weak modification (defined here as the least reactive regions) being observed. The most reactive regions were 39–56 nt, 147–158 nt, 801–806 nt, 448–458 nt, 464–470 nt, and 476–484 nt. The least reactive regions were 57–70 nt, 115–132 nt, 135–145 nt, 159–174 nt, 180–203 nt, 254–271 nt, 275–353 nt, 357–384 nt, 387–411 nt, 559–591 nt, 637–658 nt, 717–735 nt, and 759–784 nt.

### 2.2. Secondary Structure of Segment 8 vRNA in Cell Lysate

The secondary structure of segment 8 vRNA in cell lysate was predicted. SHAPE data were used as pseudo-energy constraints, while strong DMS modifications were used as chemical mapping constraints in secondary structure predictions via the RNAstructure program (Materials and Methods). We decided to use different folding approaches during the secondary structure prediction. First, we predicted Minimum Free Energy (MFE) structure without base-pairing distance constraints to analyze local motifs as well as long-distance interaction (global structure). Secondly, we performed local MFE RNA folding (local structure) using a maximum base-pairing distance. To check how the folding algorithm and approach influence the prediction, we also used the Maximum Expected Accuracy (MEA) mode of the RNAstructure program for both local and global structures [48,49].

**Figure 2:**
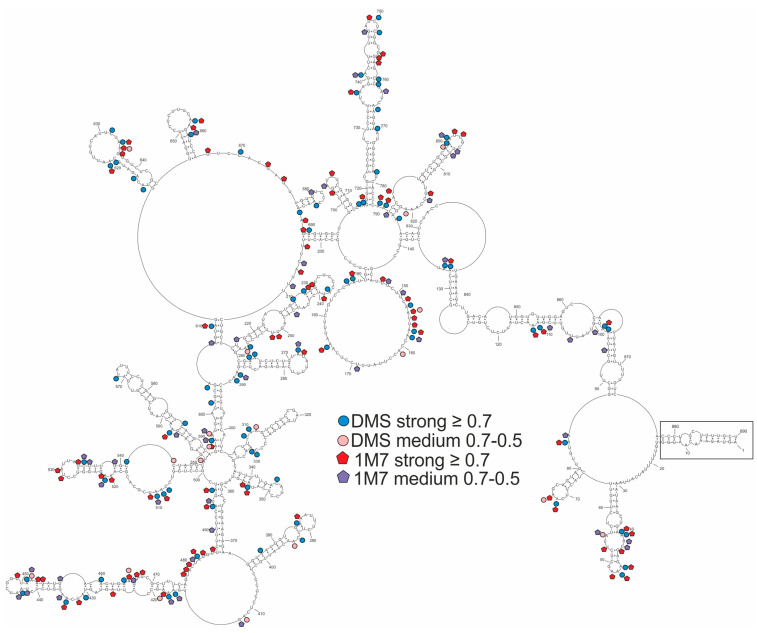
Model of the MFE secondary structure of vRNA8 A/California/04/2009. This global folding model was predicted according to chemical probing with DMS and 1M7 (SHAPE) in cell lysates. Strong (≥0.7) and medium (0.7–0.5) reactivities are marked on the structure. The *panhandle* structure between the 3′ end and 5′ end is marked. Due to the limitations of reverse transcription as well as the readout method, there are no data from 1–38 nt, 818–890 nt (1M7); and 1–13 nt, 827–890 nt (DMS).

**Figure 3:**
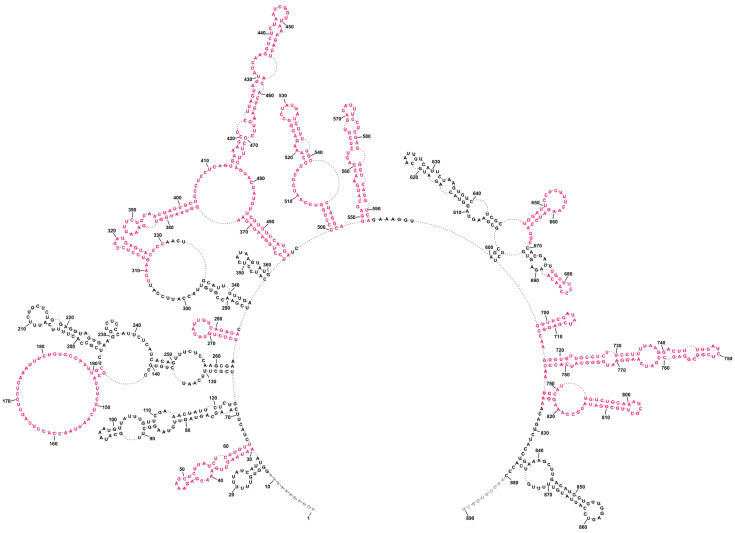
Local folding of vRNA8 A/California/04/2009 as predicted by RNAstructure using a maximum pairing distance. The structure was predicted by applying a maximum pairing distance of 150 nucleotides, with constraints from chemical probing with DMS and 1M7 (SHAPE) in cell lysates. This structure shows the preservation of 12 hairpins and a long loop region (red color) also predicted in the global structure without a distance restriction on base pairing (Figure 2).

The global approach of vRNA8 folding with no base-pairing distance restrictions resulted in a moderately structured segment encompassing both long single-stranded and highly structured regions, including 17 hairpin motifs (Figure 2). The structure was consistent with modification data with modified nucleotides located mostly in loops and other single-stranded regions. The most reactive loop region was between 146–190 nt. This global model is different from the secondary structure predicted for naked A/California/04/2009 vRNA8 [50]. The vRNA8 structure from the local approach was used to compare differences in folding between global and local structures and highlight preserved local motifs, which we treated as a confident prediction. We excluded the *panhandle* region from this prediction and limited base-pairing formation to within 150 nt with the inclusion of experimental constraints data. The predicted vRNA8 local structure (Figure 3) showed preservation of 12 of the 17 hairpins predicted in the global structure in these regions: 30–64 nt, 261–284 nt, 312–327 nt, 374–403 nt, 414–476 nt, 500–547 nt, 549–592 nt, 645–666 nt, 677–688 nt, 698–713 nt, 717–784 nt, and 787–824 nt.

**Figure 4:**
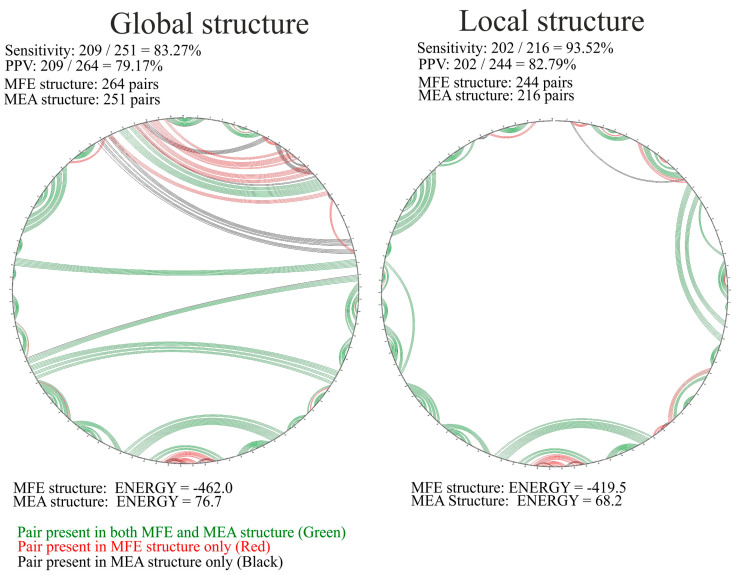
Comparison of the predicted MFE and MEA structures of vRNA8 in cell lysates. For the comparison both global as well as local structures were compared via CircleCompare tool (RNAstructure www.rna.urmc.rochester.edu, accessed 27 January 2022).

Finally, global and local MEA structures were calculated (Supplementary S1 Text: Figures S2 and S3). Both MEA structures tended to be slightly less structured when compared to MFE (Figure 4). However, the MEA local structure (Supplementary S1 Text: Figure S3) was highly similar to the MFE structure (Figure 4), as very high sensitivity and PPV (positively predicted value) values can be observed. In the case of the global structures, the sensitivity and PPV values were lower (Figure 4). These values were influenced by different structure folding in long-distance pairings in regions 97–117/876–849 nt, 136–139/829–816 nt, and 180–193/829–816 nt (Figure 4).

### 2.3. Well-Defined Structural Motifs of vRNA8 in Cell Lysate

**Figure 5:**
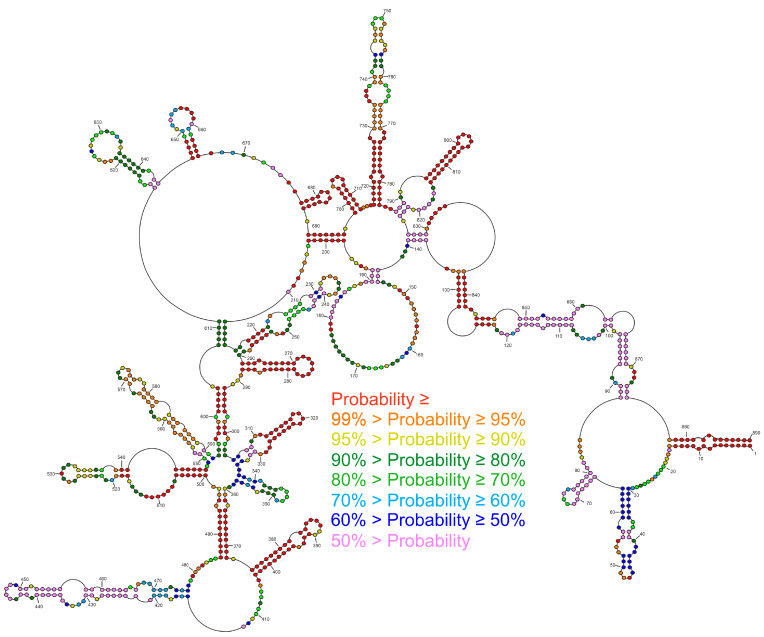
Base-pairing probabilities of single- and double-stranded regions of the vRNA8 A/California/04/2009 structure in cell lysates. The colors indicate the percentage of pairing probability. The probabilities were calculated via the partition function in RNAstructure 6.2 using experimental data constraints.

After implementation of the experimental data into the RNAstructure program, many alternative secondary structures were predicted, and those differed subtly in energy values from the MFE structure. Therefore, the probability of base-pairing of the vRNA8 structure was calculated via the partition function in the RNAstructure program. The probability calculation estimates the certainty of prediction using all predicted base pairs (BP) and unpaired nucleotides in a sequence, according to implemented experimental data. An overall high probability of the proposed vRNA8 global structure in cell lysates was calculated (Figure 5). These results indicated 10 local motifs of high (≥90%) probability that were also present in the local fold of vRNA8: 261–288 nt, 310–329 nt, 374–404 nt, 500–547 nt, 553–589 nt, 645–666 nt, 677–687 nt, 698–713 nt, 717–784 nt, and 792–814 nt. The highest probability (≥95%) of base pairing was obtained for interactions: 123–126/845–848 nt, 127–133/842–836 nt, 197–203/696–690 nt, 292–304/605–593 nt (unpaired: 297/600 and 301/596 nt) and 360–372/598–486 nt (unpaired 363/495 nt). The lowest probability was observed for long-range interaction 40–117/848–876 nt as well as in motif formed in region 414–476 nt.

**Figure 6:**
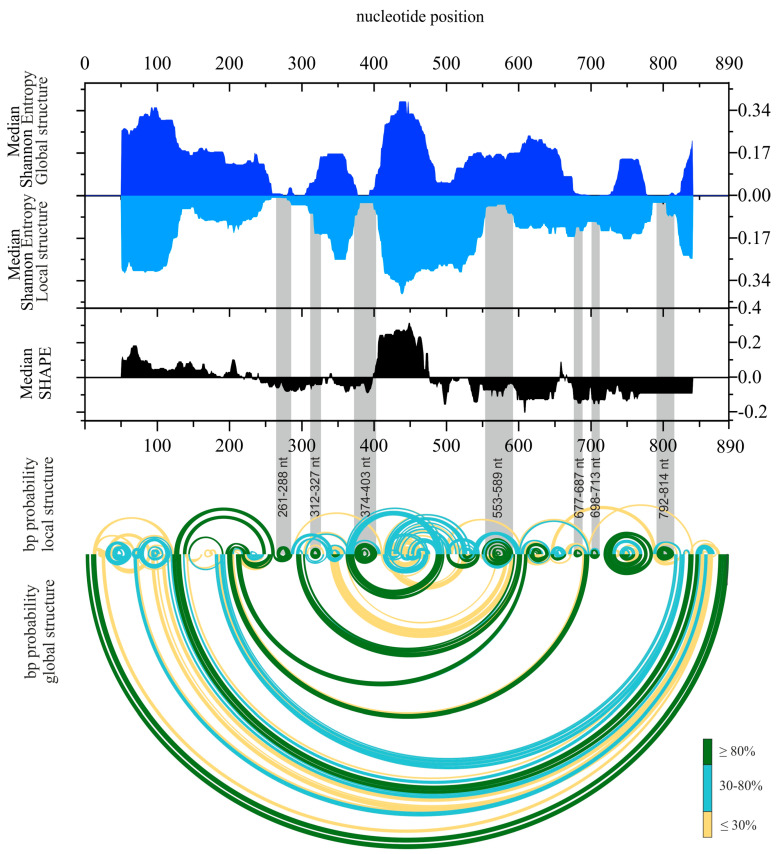
The secondary structure of vRNA8 in cell lysate. Median Shannon entropies of global and local structures were calculated in a centered, sliding 50 nt window. Median SHAPE (1M7) reactivities were calculated in 50 nt window and plotted with respect to global median. Arc plots showing the base-pairing probabilities of predicted local (upper) and global (lower) structures were estimated using the partition function (RNAstructure). Grey shadings indicate the low Shannon entropy/SHAPE reactivity regions of the most probable, well-defined structural motifs predicted in both global and local vRNA8 secondary structures. Calculations for regions of 50 nt of vRNA8 from both ends (Median Shannon, SHAPE) were excluded from visualization. No reactivity data were obtained in regions: 1–38 nt and 818-890 nt (1M7).

A Shannon entropy calculation was also used to measure the extent to which the predicted structure was well-defined. Shannon entropy quantifies the likelihood of a particular RNA fragment to form a single structure, or on the other hand, to fold to alternative conformation in equilibrium with each other. In detail, the regions indicated as having both low SHAPE reactivity as well as low Shannon entropy values in prediction formed the most likely base pairs. Seven from ten motifs with high base-pairing probabilities with low SHAPE reactivities and low Shannon entropies were in regions 261–288, 312–327, 374–403, 553–589, 677–687, 698–713, 792–814 nt (Figure 5 and Figure 6).

### 2.4. Conservation of Predicted vRNA8 Structure in Cell Lysate throughout Influenza A Strains

**Figure 7:**
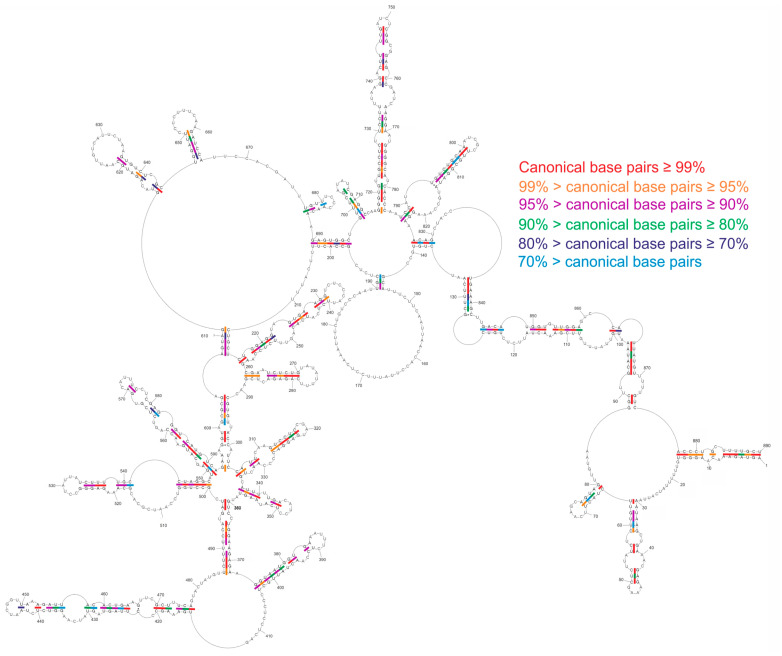
Conservation of vRNA8 global structure across type A influenza. Base pair conservations were marked based on an analysis of 34,248 IAV strains sequences obtained from the NCBI Influenza Virus Database.

**Figure 8:**
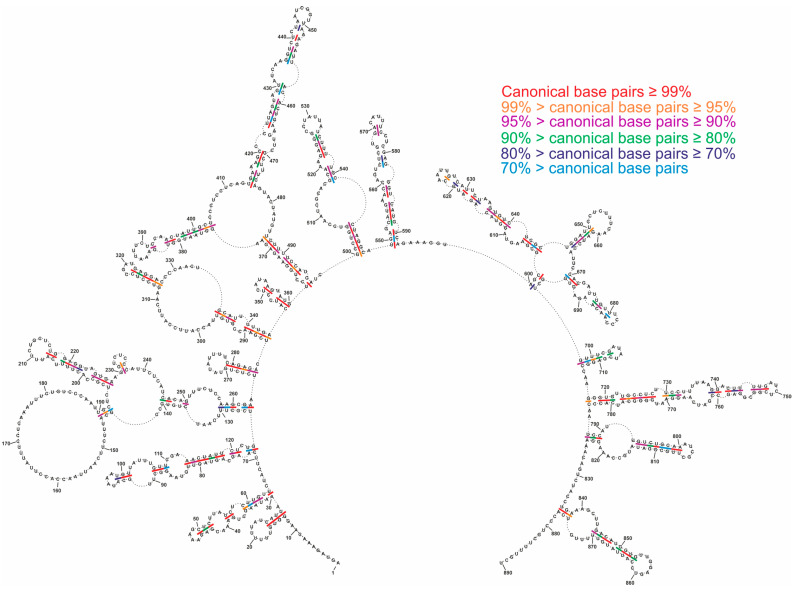
Conservation of vRNA8 local structure across type A influenza. Base pair conservations were marked based on the analysis of 34,248 IAV strains obtained from the NCBI Influenza Virus Database.

We performed a conservational analysis of local and global MFE structures based on all available vRNA8 sequences in the NCBI Influenza Database (nearly 35 thousand IAV sequences). Such analysis showed conservation of the predicted base-pairing through IAV strains. The structure conservation analysis indicated helixes of high conservation within distant IAV strains (Figure 7 and Figure 8). Interestingly, average conservations of local and global MFE structures were very similar (86.38% for global and 86.45% for local prediction). However, a more detailed analysis of both structures indicated highly conserved (≥95%) motifs predicted in both or either only in local or global folding (Table 1). A few highly conserved motifs were predicted in both local and global MFE structures in the following regions: 265–270/284–279 nt, 312–317/322–327 nt, and 719–728/773–782 nt (Table 1).

## 3. Discussion

### 3.1. Structural Probing of vRNA8 in Cellular Environment Showed Difference in RNA Accessibility to Chemical Reagents

Viral genomic RNA plays an important role during the replication cycle of the influenza virus. As of today, data concerning the IAV’s RNA structure in the biological environment are very limited. Recently, the genomic structure of A/WSN/33 strain in virio was published [29]. However, it was not clear if the predicted structure is reflected in a cellular environment as well. For that reason, we decided to examine the influence of biological crowding on the vRNA structure and we created in vivo-like conditions for the structure probing. Such an approach represents the conditions between in vitro and in vivo environments and mimics the natural conditions of RNA structure folding [51].

Cellular proteins and nucleic acids present in cell lysates could interact with vRNA8, resulting in different nucleotide reactivities and structural changes when compared to in vitro experiments. Obtained results agree with the assumption that chemical probing of vRNA8 in cell lysates would result in a different chemical mapping profile than in vitro. Indeed, we observed increased reactivities of vRNA8 nucleotides (both versus DMS and 1M7) probed in cell lysates than during in vitro probing [50]. In comparison, the in virio vRNA structures in vRNP complexes of A/WSN/33 (H1N1) strain also showed increased SHAPE reagent reactivity across the vRNAs when compared to naked vRNA [29].

### 3.2. Different RNA Structure Prediction Approaches Identified Structural Motifs with High Fidelity

Three different approaches were used for vRNA secondary structure folding using all experimental data. First, to predict long-range interactions, the structure was folded globally without restrictions in the maximum distance of the base pairing. The advantage of global folding is the identification of potential distant interactions, potentially crucial during the viral replication cycle. An example of the long-range interaction is a well-known *panhandle* duplex structure forming between the 3′ and 5′ ends of vRNA which serves as a viral polymerase promoter [52]. A recently published high-resolution structure of vRNA in virio confirmed the formation of this *panhandle* structure [29]. Importantly, this region is highly conserved across all vRNA segments within all IAV strains. Moreover, the *panhandle* structure is important for recognition by RIG-I and thus IFN production [53]. Therefore, we decided to include the base pairing in the *panhandle* region of vRNA8’s final structure (region 1–11/881–890 nt) during the prediction of the global vRNA8 structure.

Analysis of long RNAs in a biological context is very complex, as low-reactivity regions could be either RNA double-stranded regions or regions involved in RNA–protein interactions. During the influenza replication cycle, vRNA interacts with viral as well as cellular environmental components. In a biologically active environment, the locally folded motifs are easier to distinguish. For that reason, we also used a second approach during the folding. Namely, the structure was folded locally by limiting the base-pairing distance to 150 nt. The same approach of structure modeling was used in the case of vRNAs of A/WSN/33 strain structure folding using data from chemical mapping in virio [29]. Using the same folding parameters allowed us to compare the vRNA8 structure in cell lysate with published in virio structures.

The last approach was the prediction of a model secondary structure via the MaxExpect algorithm in RNAstructure (MEA structure) [54]. Notably, MaxExpect constructs the overall secondary structure by maximizing the single-stranded and pairing probabilities extracted from a predicted partition function [48,55]. We included experimental data (SHAPE and DMS constraints) in the partition function calculation to glean experimentally informed models of the most probable configurations predicted. Using different approaches during the structure folding in the RNAstructure program showed the differences in the prediction depending on the used constraints and algorithm (Figure 2 and Figure 3, Figures S2 and S3). For that reason, special caution should be taken during the interpretation of the structural data. For a more accurate interpretation of the data, different folding parameters should be used and should consider additional constraints. The comparison of the predictions (MFE, MEA) indicated well-determined structural motifs, which were predicted independently from the used method.

### 3.3. Several Well-Defined and Conserved for IAV Structural Motifs of vRNA8 Were Identified

We found 10 structural motifs of high (>90%) base-pairing probability predicted in all: MFE, MEA, and both local and global structures (Figure 4). We also found a few long-distance interactions with very high (>95%) base-pairing probabilities. The partition function calculation was further used for the calculation of the Shannon entropy values. The comparison of the low Shannon entropy values with the median values of SHAPE reactivity enabled us to distinguish the regions of low Shannon entropy–low SHAPE reactivity values. These are the most probable to form one well-defined structural motif. Among the 10 high-probability motifs, 7 were predicted in these regions.

**Figure 9:**
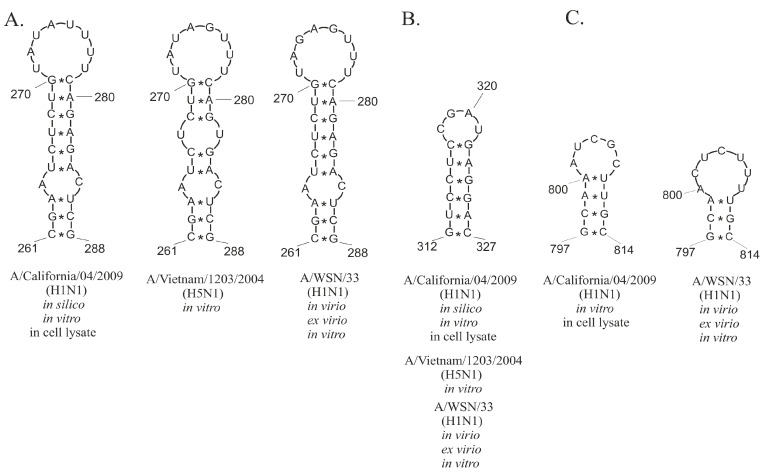
Conserved structural motifs of vRNA8 within different IAV strains. The motifs were predicted based on bioinformatics analysis and chemical probing experiments in different conditions. (**A**) Motif 261–288 nt, (**B**) Motif 312–327 nt, (**C**) Motif 797–814 nt.

Additional support for the functional existence of certain motifs is conservation despite sequence changes in different IAV strains. We identified some motifs with high conservation predicted exclusively either in the local or global MFE vRNA8 structures. We also identified a few structural motifs of very high (≥95%) base-pairing probability that were predicted in both local and global MFE structures (Table 1). In particular, three highly conserved motifs in regions 265–284 nt, 312–327 nt, and 719–782 nt were predicted independently of the folding approach used (Figure 9).

Little was previously known about vRNA structures in a biological environment; however, recently published data of vRNA chemical mapping and structures in vRNP complexes ex virio and in virio of the A/WSN/33 strain changed this situation [29]. A/WSN/33 and A/California/04/2009 are distant strains with low vRNA8 sequence similarity (85, 2%, CLUSTALW). The authors of the A/WSN/33 study focused on local folding in virio, forcing a maximum pairing distance of 150 nt. Using the same folding constraints in RNAstructure against the A/California/04/2009 vRNA8 cell lysate data, it is possible to compare the two strains. Common structural motifs were observed in the following regions: 261–288 nt (Figure 9A), 312–327 nt (Figure 9B), and 792–814 nt (Figure 9C), and their structural conservation was 97.22, 96.09, and 87.61%, respectively. Notably, these three motifs were also predicted in global structure (Figure 6). The conservation of these motifs within different IAV strains in different probing environments is another suggestion that these could play some functions during the viral replication cycle. For example, the motifs might potentially take part in vRNA–vRNA or vRNA–protein interactions. Comprehensive research of vRNA–vRNA interactions showed the complexity of such networks [29]. The first models of packaging signals suggested that only ~50–150 nt of both vRNA termini are engaged in virion packaging [56]. Dadonaite et al. confirmed a different model of packaging signals and showed that these might occur distant from termini ends [29]. These data suggest that revealed conserved motifs might be packaging signals (Figure 9).

### 3.4. Comparison of vRNA8 Structure in Cell Lysates and In Vitro

**Figure 10:**
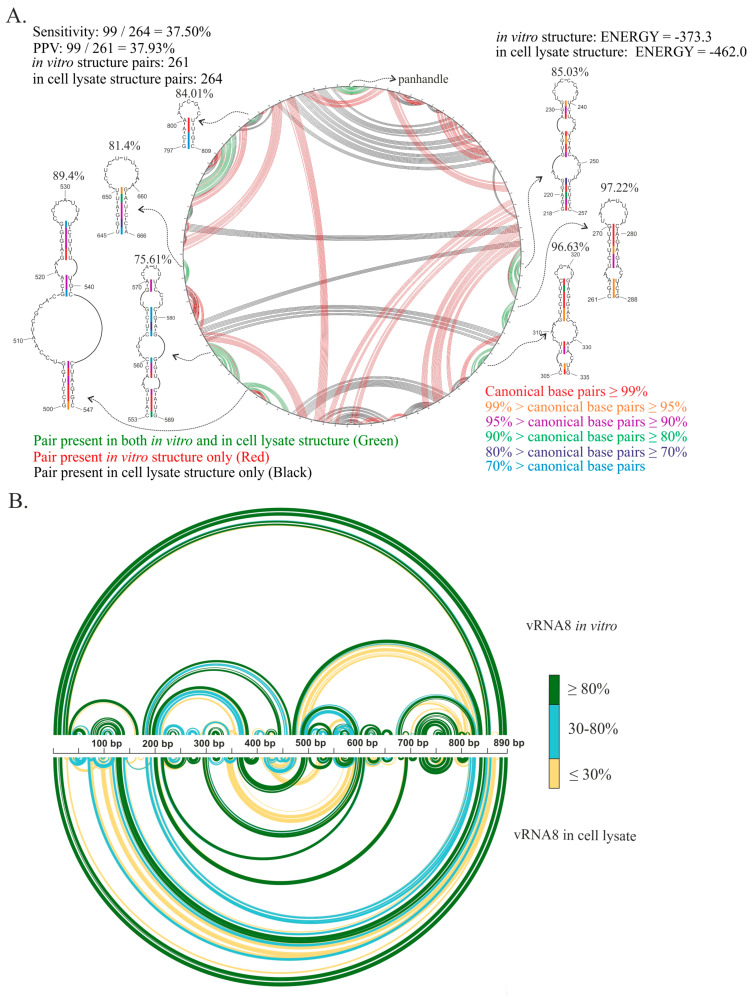
Comparison of vRNA8 A/California/04/2009 structures predicted based on experimental results in vitro and in cell lysates. (**A**) CircleCompare comparison of structures shows differences (red color—pair predicted in vitro, black color—pair predicted in cell lysate) and similarities (green color) in base-pairing of vRNA8 between in vitro and in cell lysate. Low sensitivity and PPV values indicated a low resemblance between the vRNA8 structure probed in different environments. Structural motifs predicted in both environments are shown and the base-pairing conservation according to a color scale and average percentage conservation of each motif is indicated above. (**B**) Comparison of vRNA8 structures predicted in vitro and in cell lysates with the base-pairing probabilities calculated with partition function (RNAstructure). The colors indicate the probability of each base pair in a three-point color scale.

Our previous study concerning the secondary structures of the vRNA8 of A/Vietnam/1203/2004 (H5N1) and A/California/04/2009 (H1N1) in vitro showed differences between the strains, but a few common structural motifs were predicted in those evolutionally distant strains [33,50]. To investigate which structural features are preserved or changed in a biological context, we compared the vRNA8 of A/California/04/2009 (H1N1) MFE global structure in cell lysate and in vitro (Figure 10). The CircleCompare comparison of both structures showed very low sensitivity and PPV values (37.5% sensitivity, 37.93% PPV) (Figure 10A) indicating vRNA8 structures vary between the probing environments. The highest differences were observed in the long-range interactions (Figure 10A,B). Nonetheless, we found seven structural motifs common for both structures (Figure 10A). The conservation of each motif within all IAV strains showed that most of them are moderately conserved (Figure 10A).

The base-pairing probabilities based on partition function calculations (RNAstructure) showed that the vRNA8 structure in lysate is more variable in the 3‘ end and region ~410–480 nt when compared to in vitro structure. A few structural motifs exclusive for vRNA8 in cell lysate had high base-pairing probabilities in the following regions: 374–403 nt, 677–687 nt, and 689–713 nt. These motifs were observed in regions indicated as low Shannon entropy/low SHAPE reactivity regions (Figure 6).

Notably, three motifs in vRNA8 regions 218–257 nt, 261–288 nt, and 305–335 nt in cell lysate structure were predicted for the A/Vietnam/1203/2004 in vitro vRNA8 structure and in the consensus vRNA8 structure of IAV [33]. That indicates the importance of conducting comparative research of the vRNA structures within different IAV strains. Moreover, three motifs in regions 261–288 nt, 305–335 nt, and 645–666 nt were also predicted in silico for the A/California/04/2009 strain [34]. This, in turn, points to the usability of in silico and in vitro studies for secondary structure prediction. However, further study for confirmation of the presence of such structural motifs in the biological context must be performed.

### 3.5. Motifs of the Highest Base-Pairing Probability Are Present in NP-Enriched and NP-Depleted Regions of vRNA in vRNP Complexes

Research concerning protein–vRNA interactions in vRNP complexes showed that NP is non-uniformly bound to vRNA as regions rich and poor in such association can be distinguished [38]. The properties of the structure of vRNA8 in the cellular environment were compared to an NP-binding profile within a vRNP complex of the same strain [38]. An NP-binding profile showed the regions between 1–295 nt, 400–520 nt, 695–720 nt, and 855–890 nt to be relatively less bound to NP or almost NP-free, while moderate NP binding was observed in regions between 295–400 nt, 520–555 nt, 630–695 nt, and 720–775 nt [38]. These regions might be crucial for the intersegmental vRNA–vRNA or vRNA–protein interactions. Extensive NP binding was observed in regions between 555–630 nt and 750–855 nt. Interestingly, we observed that vRNA8 structural motifs with a high probability (>90%) of base pairing (Figure 5) were predicted in both low-NP and high-NP associated regions [38]. Additionally, several motifs with >90% conservation (261–288 nt, 312–327 nt, and 500–547 nt) were low in NP binding, whereas two motifs that had the highest abundance of NP (553–589 nt and 792–814 nt) had low conservation in IAV (77.8 and 81.78%, respectively).

NP-binding profiles for the A/WSN/33 and A/California/04/2009 strains are unique to both strains [38]. Conservation in secondary structure motifs constrained natural changes in the genome between strains and could influence NP binding. Three recognized conserved motifs between the two strains are 261–288 nt, 312–327 nt, and 797–814 nt. Despite the conserved motifs, the NP binding between these strains varies greatly in these regions [38]. Dadonaite et al. concluded that RNA secondary structures in virio are slightly impacted by RNA–NP interactions [29]. The presented research agrees with this general statement. The presence of conserved motifs in both strains is not directly dependent on NP-binding properties [38].

### 3.6. vRNA8 Structure Accessibility in a Cellular Environment Could Be Useful to Design Antivirals

Knowledge concerning secondary structures of the viral RNAs not only brings us to a better understanding of viral biology but has a practical dimension as well. It enables the design of viral inhibitors directly targeting RNA, as their activity depends on the accessibility of a particular RNA region [57]. The development of such technology is especially important, as more and more drug-resistant influenza strains evolved with time [58]. Different RNA-based nucleic acid therapies have been proposed in the case of RNA viruses [59]. Depending on the mechanism of the inhibition, these can block the replication processes of the virus by creating steric blockades or lead to RNase H cleavage of targeted RNA [57]. It must be pointed out that depending on the used method, it demands information about the specific structural features of the targeted RNA region. One of the common types of RNA-targeting synthetic molecules are antisense oligonucleotides (ASOs) [57,60].

We found that the effectiveness of ASOs against influenza vRNA was variable, indicating that some regions were not accessible for inhibition in cells [31,34]. Various short 2′O-methylated RNA and locked nucleic acids (LNA) antisense oligonucleotides targeting vRNA8 of the A/California/04/2009 strain were tested previously [34]. Five of these oligonucleotides exhibited significant inhibitory properties against the influenza virus: 404-14L (GA^L^GGA^L^GGGA^L^GCA^L^AU), 187-14L (GA^L^GCA^L^AU^L^UGGGA^L^CA), 68-11L (UU^L^GAA^L^GU^L^GGA^L^A), 167-15L (UGA^L^GGA^L^AAU^L^AAGGU^L^G), and 867-14L (CA^L^AA^L^AA^L^CA^L^UA^L^AUGG), which lowered viral titer by 1.4, 1.2, 1.3, 0.7, and 0.7 log_10_[FFU/mL], respectively. They targeted the following regions: 398–411, 181–194, 63–73, 160–174, and 861–874 nt, respectively. In the context of the availability of vRNA8 complementary regions to these oligonucleotides in the cellular environment, the inhibition results are mostly in agreement with the determined herein secondary structure of vRNA8. The target regions fold the same in local and global vRNA8 structure prediction, except at the target region for 867-14L. The 867-14L binding site base-pairing probability is low in MFE (Figure 5), but conserved (Figure 7), whereas the MEA structure (Supplementary S1 Text: Figure S3) has the same folding in this region as in silico structures used in the design of the oligonucleotides [34]. Interestingly, the vRNA8 target regions for 187-14L (181–194 nt) and 167-15L (160–174 nt) in cellular lysate are long, single-stranded regions.

Analysis of the correlation between the effectiveness of particular ASOs and the secondary structure of the vRNA8 in cell lysate led us to an interesting observation. ASOs that caused significant IAV replication inhibition targeted the regions in which the median Shannon entropy and SHAPE values were high, while the base-pairing probability was low (Figure 6) [34]. High Shannon entropy indicates a lower probability that well-defined structural motifs are formed in that regions. The regions predicted to be more variable might be affected by different kinds of interactions such as intra- or intersegmental vRNA interactions, interactions with cellular RNAs, or interactions with the viral or cellular proteins [25,29,38]. Notably, such interactions may disturb the chemical probing and thus the structure prediction. In addition, the regions of high SHAPE reactivity are predicted to be more single-stranded, thus might be potentially accessible for the ASOs targeting. Such considerations led to the assumption that the effectiveness of each ASOs is dependent on the outcome of many events. All of that makes the design of antisense therapy a very complex issue, as one simple explanation of mechanisms that are interrupted by ASOs cannot be proposed.

Another promising inhibitory strategy targeting viral RNA is RNA cleavage catalyzed by the CRISPR/Cas13 system guided by short guide RNAs (gRNA) [61,62]. Notably, the secondary structure of the RNA also affects the CRISPR/Cas13 system, as it targets the single-stranded RNA [63]. The system was already tested in the case of the SARS-CoV-2 and influenza viruses [62,64]. However, the authors observed variable efficiency in the viral replication inhibition, which was probably affected by the inaccessibility of targeted regions due to the RNA structure or possible RNA–protein interactions [62,64]. That indicates that the secondary structure of target RNAs must be considered for the design of gRNAs [65]. The structural data and analysis provided herein could be useful for designing more effective anti-influenza CRISPR/Cas13 systems.

Influenza virus and its fast evolution brings us to the point that the most effective and universal antiviral strategy should be based on the most conserved RNA structural elements. The aforementioned strategy’s success relies on accessibility of the target regions for base pairing. Effective inhibitors targeting double-stranded RNA (dsRNA) regions also depend on knowledge about RNA structure and accessibility. An example might be peptide nucleic acids (PNAs) which can form a triple helix with targeted dsRNAs [57]. Modified PNA targeting conserved double-stranded *panhandle* structures appeared to be an efficient and specific anti-influenza inhibitor [66].

Small molecules (SMs) targeting RNA are another promising antiviral approach [67]. Specific and tight binding of SMs depend on RNA structure and many promising approaches for using SMs against pathogenic RNA have already been tested [67,68,69]. For designing SM strategies against influenza, the presented knowledge of vRNA secondary structure in cellular environments may be crucial. Thus, conserved structural motifs confirmed to occur in the vRNA8 structure predicted in a cell lysate are a great candidate for the inhibition of IAV.

## 4. Materials and Methods

### 4.1. Cell Culture and Virus Propagation

All experiments concerning the virus were performed in Madin Derby Canine Kidney (MDCK) cell line (Merck, ECACC). Cell cultures were maintained in Dulbecco’s Modified Eagle’s Medium (DMEM) supplemented with 10% heat-inactivated fetal bovine serum (FBS), 2 mM glutamine, and antibiotics (100 U/mL penicillin and 100 µg/mL streptomycin). The culture was incubated in a 5% CO_2_ environment at 37 °C.

Before infection, MDCK cells were seeded at a density of 3 × 10^6^ per 10 cm^2^ and grown to reach 100% confluency. Next, the cells were overlaid with the A/California/07/2009 (H1N1) viral dilution in the infection medium (0.3% BSA, 100 U/mL penicillin, 100 µg/mL streptomycin, in PBS) at 0.01 MOI (multiplicity of infection). After an hour, the viral dilution was aspirated, and the post-infection medium (0.3% BSA, 100 U/mL penicillin, 100 µg/mL streptomycin, 2 mM glutamine, and 1 µg/mL tosyl-sulfonyl phenylalanyl chloromethyl ketone (TPCK)-treated trypsin, completed with DMEM) was added and cells were kept at 33 °C, 5% CO_2_. After 48 h, the viral inoculum was harvested, aliquoted, and kept frozen at −80 °C before titration. Virus titration was performed with standard IFA (Immunofluorescence Focus Assay) [70]. For the infection, a 10-fold serial dilution of the virus was added to the cell culture and incubated in a post-infection medium that took 8–10 h. The cells were fixed (4% formaldehyde), permeabilized (0.5% Triton X-100 in PBS), and overlaid with blocking buffer (3% BSA) before staining. For staining, the cells were overlaid with primary antibody targeting NP (MAB8257, Merck) for 1–2 h of incubation, followed by a 1 h incubation with the secondary FITC-conjugated rabbit anti-mouse IgG antibody (AP160F, Merck) for detection. Next, the viral titer was calculated after visualization under the fluorescent microscope.

### 4.2. Preparation of the Cellular-Viral Lysates

The infection of the MDCK cells was carried out as described above, with the difference being that the cells were incubated in the post-infection medium for 24 h before the lysis. Next, the cells were washed twice with 1xPBS and incubated with 6 mL of cell lysis buffer prepared according to a published protocol [71]. After the complete lysis was confirmed under the microscope, lysates were immediately aliquoted and kept frozen at −80 °C until needed for chemical mapping experiments. Lysates from infected MDCK cells were checked for the presence of viral proteins by Western blot analysis (Supplementary S1 Text: Figure S1). The level of infection was additionally evaluated by vRNA concentration calculation via RT-qPCR (Supplementary S1 Data: Table S1; Supplementary S1 Text: Figure S4). Next, chemical probing was performed as described below. Additionally, before reverse transcription, the isolated RNA was checked for integrity on a non-denaturing agarose gel which verified an intact total RNA and single vRNA8 product (890 nt long). Three independent biological replicates were performed, meaning independent infection of the cells and cell lysate preparation.

### 4.3. In Vitro Transcription of vRNA8

Templates for the in vitro transcription were PCR-amplified from the pUC19 plasmid, which contained the complete vRNA8 sequence, constructed previously [50]. The PCR reaction was performed with Q5 High-Fidelity polymerase (NEB) using FC8 and RC8 primers (Supplementary S1 Data: Table S1) containing a sequence of T7 polymerase promoter, according to the manufacturer’s protocol in 25 µL. 150 ng of pUC19 plasmid was used per reaction.

For the in vitro transcription of vRNA8, 1 µg of template PCR product was used. The reaction was performed according to the manufacturer’s protocol (AmpliScribe T7-flash, Lucigen, Middleton, WI, USA). The RNA was cleaned on an RNeasy MinElute column (QIAGEN, Hilden, Germany) and checked for integrity on a 1xTBE 1% in the presence of RNA marker (RiboRuler High Range RNA ladder, Thermo Fisher Scientific, Waltham, MA, USA).

### 4.4. Primers Synthesis and Fluorescent Labeling of the Primers

All the oligonucleotides used in this study were synthesized in the MerMade12 synthesizer according to established procedures [72,73]. After solid-phase synthesis with phosphoramidites, the oligonucleotides designed for PCR, qPCR, and reverse transcription (RT) were deprotected as described previously [31,74]. The primers for primer extension were labeled with fluorophores (5-FAM, 6-TAMRA, 6-JOE, and 5-ROX; Anaspec, Fremont, CA, USA) according to our established procedures [31,75]. All the oligonucleotides were purified on 8M urea 12% PAGE. All primer sequences are available in Supplementary S1 Data: Table S1.

### 4.5. Chemical Probing

Cell lysates were thawed on ice and 180 µL of lysate supplemented with RNase Inhibitor (5U) were used per reaction. Briefly, 5 pmol of in vitro transcribed vRNA8 was heated for 5 min at 65 °C, cooled to 37 °C, and immediately added to the lysate for 30 min incubation at 37 °C. For the probing reaction, two types of chemical reagents were chosen—DMS (Merck, Darmstadt, Germany) and 1M7 (SHAPE), the concentrations of which were determined empirically. The 1M7 was synthesized according to published protocols [76]. The chemical reagent was used in a 1:10 ratio in the total volume of 200 µL of the final reaction. For the SHAPE probing, 250 mM of 1M7 (25 mM final) diluted in anhydrous DMSO was used and the reaction was allowed to proceed for 5 min at 37 °C. For DMS probing, the lysate was treated with 4% DMS (0.4% final) diluted in ethanol; the reaction proceeded for 15 min at room temperature and was quickly quenched by adding b-mercaptoethanol (0.04 M final). The control reaction was treated with DMSO only or ethanol, respectively. After probing, the RNA was purified on RNeasy MinElute column (QIAGEN, Hilden, Germany) and treated with DNase I enzyme (0.2 U final) followed by the ethanol precipitation. All the probing experiments were performed in three biological replicates and a minimum of three technical replicates. Reactivity data from different probing experiments were combined and the average reactivities were calculated.

### 4.6. Primer Extension by Reverse Transcription

The concentration of RNA was measured (NanoDrop 2000 Thermo Fisher Scientific, Waltham, MA, USA) and separated in 1%/1xTBE agarose gel electrophoresis. After the confirmation of RNA quality, the reverse transcription (RT) reaction was performed. RT was conducted with SuperScript IV (Thermo Fisher Scientific, Waltham, MA, USA) according to the manufacturer’s protocol with some modifications. Briefly, the 30 µL of the final reaction was combined with 16.5 µL of RNA, 1xFS buffer, 0.5 mM dNTP (EurX, Gdansk, Poland), and 2 µM of fluorescent-labeled primer (JOE for reaction, FAM for control) (Supplementary S1 Data: Table S1) and incubated for 3 min at 90 °C, 10 min at 55 °C, and kept on ice for 3 min before adding a mix consisting of 1xFS buffer, 5 mM DTT, 2 U RNase inhibitor (Promega, Madison, WI, USA), and 10 U of SuperScript IV. The reaction was incubated for 20 min at 55 °C, inactivated for 10 min at 80 °C, and cooled to 4 °C. For the RNA hydrolysis, 5 µL of 2 M NaOH was added and the reaction was incubated for 5 min at 95 °C and neutralized by adding an adequate amount of 1 M HCl. Finally, the cDNA was precipitated and the modification reaction, along with the control reaction and ddNTP ladders, were mixed and evaporated on SpeedVac (Labconco, Kansas City, MO, USA). Next, the sample was dissolved in water and separated by single-capillary electrophoresis (CE) (Hitachi Applied Biosystems 3100 Avant, Laboratory of Molecular Biology Techniques at Adam Mickiewicz University in Poznan, Poland).

### 4.7. DNA Sequencing Ladders

DNA sequencing ladders were synthesized according to the manufacturer’s protocol with modifications (USB Thermo Sequenase Cycle Sequencing Kit, Affymetrix, Santa Clara, CA, USA). Briefly, for the sequencing reaction, 90 ng of a template (PCR product) was mixed with 5 pmol of labeled primer, one of the 2′-3′-dideoxy-NTP (ddGTP for ROX, ddATP for TAMRA), 0.5x reaction buffer, and 2U of Thermo Sequenase DNA polymerase. The reaction was PCR-amplified and ethanol-precipitated.

### 4.8. Real-Time PCR

As a reference standard for the quantitative PCR the vRNA were used. After vRNA in vitro transcription from pUC19 plasmids, the standards were reverse transcribed using SuperScript III according to the manufacturer’s protocol, and serial 10-fold dilutions were prepared. The primers matching short fragments of chosen vRNAs (7VRT or 8VRT; Supplementary S1 Data: Table S1) were designed according to published protocols [77,78]. The RNA from lysates was reverse-transcribed with SuperScript III enzyme according to the manufacturer’s protocol. The qPCR reaction was performed using 5 µM VTAG and 5 µM VQR (7VQR, 8VQR), and 1x Hot FIREPol^®^EvaGreen^®^ qPCR Mix (no ROX) (Solis Biodyne, Tartu, Estonia). All the samples were set up in triplicate, and negative control of total RNA from mock-infected MDCK cells was used.

### 4.9. Western Blot

24 h after infection with IAV (MOI 0.01), cells were lysed with lysis buffer (60 mM Tris pH 6.8, 10% glycerol, 2% SDS, 5% β-mercaptoethanol, 0.1% Bromophenol blue, and 1 mM EDTA). The total protein in cellular lysates was stained with Coomassie Brilliant Blue R-250 Staining Solution (Bio-Rad, Hercules, CA, USA). Additionally, IAV proteins expression was detected by Western blot analysis using rabbit anti-IAV NP (PA1-41071, Thermo Scientific) and mouse anti-IAV HA (SAB2702266, Merck Millipore) antibodies with HRP-coupled anti-rabbit (AP307P, Merck Millipore) and anti-mouse secondary (12-349, Merck Millipore) antibodies, respectively. Expression of actin, detected with mouse anti-actin monoclonal antibody (ACTN05(04), Invitrogen) and anti-mouse HRP-conjugated antibodies (12-349, Merck Millipore), served as a loading control (Supplementary S1 Text: Figure S1).

### 4.10. Chemical Mapping Data Analysis

The results of capillary electrophoresis in ABI format were analyzed with ShapeFinder software [79]. To overcome differential migration, which is typical for the various fluorophores separated in single-capillary electrophoresis, additional calibration (Mobility Shift tool) was performed. The area of analysis was limited to contain only intact data (Signal Decay Correction tool) and adjusted with a Scale Factor tool if needed, and all results were fully aligned. The file consisting of final nucleotides’ reactivities was later normalized via a 2–8% normalization method [31,80]. Reactivities ≥ 0.7 were considered as strong, 0.7–0.5 were considered as a medium, and ≤0.5 as weak. Each probing experiment (DMS, 1M7) and data analysis were made independently in at least three replicates. For the final reactivity file, the average reactivity of each nucleotide was calculated. Nucleotides with unknown reactivity data were indicated as −999.

### 4.11. Secondary Structure Prediction and Visualization

The global secondary structure prediction of vRNA8 was generated using RNAstructure Version 6.2 [81,82,83]. As a constraint, a SHAPE data file containing average reactivities from replicates was used with a slope of 1.8 and an intercept of −0.6 kcal/mol. Subsequently, the file containing strong DMS reactivities and base pairing in promoter structure (*panhandle*) was introduced. For the local folding prediction, a maximum pairing distance was implemented at 150 nt, and the promoter structure region was excluded from the structure folding.

The predicted structures were converted to dot-bracket files in RNAstructure and visualized in PseudoViewer2.5 [84]. For the base-pairing probability calculations, the partition function was used and data from chemical mapping (SHAPE and DMS constraints) were implemented. Generated dot plot files were visualized with IGV (version 2.8.9) [85]. The maximum expected accuracy structures were calculated with the MaxExpect algorithm (RNAstructure) [54] according to the probability calculation file.

### 4.12. Bioinformatic Analysis of Structure Conservation

All available vRNA8 sequences were obtained from the NCBI Influenza Virus Database. The selection criteria were nucleotide sequence, influenza A virus type, segment 8 (MP), full-length only. This resulted in 34,248 IAV sequences. The coding RNAs were then reverse transcribed using Biopython’s reverse complement function and aligned using MAFFT (FFT-NS-1 method). Custom scripts were then used to map and align vRNA8 motifs to these alignments as well as count the occurrences of nucleotides at each base pairing location. Conservation of base pairing was calculated to give the percent conservation of canonical base pairing (GC, AU, and GU pairing), as well as a measure of inconsistent, or potentially non-canonical, pairing. Although rare in full-sequence databases, ambiguous nucleotides (such as N for any) were considered non-canonical in conservation calculations. The inclusion of these base pairings may result in slightly higher calculated levels of conservation but not in a way that would be considered statistically relevant.

### 4.13. Calculations of Shannon entropy

The per nucleotide Shannon entropy was calculated using the following:

(1) $$S_{i}=-\overset{N}{\underset{j=1}{\sum }}P_{i,j}\;\cdot \;\log_{10}\left(P_{i,j}\right)$$

where *S_i_* is the Shannon entropy for nucleotide *i*, *P_i,j_* is the probability of base pairing for nucleotides *i* and *j*, and *N* is the sequence length. Probabilities are calculated using the partition function in RNAstructure [81], incorporating experimental mapping data in the calculation. Shannon entropies reflect the well-definedness of secondary structure formation and are reduced when structure prediction is guided by experimental mapping data [86,87].

## 5. Conclusions

Information about vRNA secondary structure in the cellular environment is highly anticipated for better understanding of influenza biology and designing therapies. At the same time, challenges in conducting and interpretation of in-cellular RNA structural probing create a need for a simpler, in vivo-*like* system to gain valuable information that further could also be used for better interpretation of in-cell data. Here, we used such in vivo-*like* cellular environment conditions for vRNA of IAV.

The secondary structure of vRNA8 of the A/California/04/2009 (H1N1) strain folded in the presence of the cellular and viral components was analyzed and predicted based on chemical mapping data. A multipronged folding approach allowed us to highlight important and highly probable motifs. vRNA8 prediction in RNAstructure and experimental data with and without pairing distance restriction showed different folding when compared to the in vitro structure. Interestingly, the highest calculated probabilities for single- and double-stranded regions of vRNA8 structures in cell lysate were obtained in seven motifs predicted also in vitro for the same strain. A total of 12 of 17 predicted hairpin motifs were present in both global and local approaches with base-pairing distance restrictions. The comparison of Shannon entropy and SHAPE reactivity allowed us to distinguish seven well-defined motifs: 261–288, 312–327, 374–403, 553–589, 677–687, 698–713, and 792–814 nt. Three motifs, 216–260 nt, 261–287 nt, and 312–327 nt, were predicted before for the distant IAV strain A/Vietnam/1203/2004, while the motifs 312–327 nt, 500–547 nt, 645–666 nt were predicted in silico. The motifs 261–288, 312–327, and 797–814 nt are also found for vRNA8 in virio and ex virio of various IAV strains. Importantly, the conducted study also indicated accessible regions of vRNA8 in cellular environment. The previous research showed that the best antisense oligonucleotides targeted, confirmed herein, unstructured and accessible vRNA8 regions: 398–411, 181–194, 63–73 and 160–174 nt.

Structure conservation calculations for predicted vRNA8 motifs in a cellular environment revealed high levels of conservation across many IAV strains. The conserved RNA motifs appear to fold independently of regional nucleoprotein-binding affinity, but a low or uneven distribution of nucleoprotein (NP) in each motif region is noted. Some of the motifs were predicted exclusively in either global or local predicted structures. That indicated the importance of considering multiple approaches during the structure prediction, as some important structural features might be omitted by limiting the prediction to only one folding method. The presented research reveals conserved motifs that may play a crucial role during the viral replication cycle. The vRNA8 conserved motifs and knowledge of vRNA8 accessibility could be used for universal anti-viral therapy based on RNA structure.

## Figures and Tables

**Table 1:** The motifs with the highest, ≥95%, conservation of the base-pairing within IAV, predicted in local and global MFE structures. The motifs predicted in both local and global structures are bolded.

Global MFE vRNA8 Structure		Local MFE vRNA8 Structure	
motif	conservation of base pairs	motif	conservation of base pairs
1–15/890–877	98.13%	13–16/28–25	99.6%
216–231/260–240	95%	**265–270/284–279**	97.02%
261–270/288–279 including **265–270/284–279**	97.22%	286–317/344–322 including **312–317/327–322**	97.28% 96.09%
305–317/335–322 including **312–317/327–322**	96.63% 96.09%	345–350/360–355	99.81%
**719–728/782–773**	96.53%	**719–728/782–773**	96.53%

## Data Availability

The data presented in this study are available in the article and its Supplementary Materials.

## Supplementary Materials

The following supporting information can be downloaded at: https://www.mdpi.com/article/10.3390/ijms23052452/s1.

## References

[1] Su S., Fu X., Li G., Kerlin F., Veit M. (2017). Novel Influenza D virus: Epidemiology, pathology, evolution and biological characteristics. Virulence.

[2] Mostafa A., Abdelwhab E.M., Mettenleiter T.C., Pleschka S. (2018). Zoonotic potential of influenza A viruses: A comprehensive overview. Viruses.

[3] World Health Organisation (2019). Global Influenza Strategy Summary 2019–2030 Influenza.

[4] Francis M.E., King M.L., Kelvin A.A. (2019). Back to the future for influenza preimmunity—Looking back at influenza virus history to infer the outcome of future infections. Viruses.

[5] Duwe S. (2017). Influenza viruses-antiviral therapy and resistance. GMS Infect. Dis..

[6] Houser K., Subbarao K. (2015). Influenza vaccines: Challenges and solutions. Cell Host Microbe.

[7] States U. (2010). CDC Estimates of 2009 H1N1 Influenza Cases, Hospitalizations and Deaths in the United States, April–December 12, 2009. Emerg. Infect. Dis..

[8] Kim H., Webster R.G., Webby R.J. (2018). Influenza Virus: Dealing with a Drifting and Shifting Pathogen. Viral Immunol..

[9] Shao W., Li X., Goraya M.U., Wang S., Chen J.L. (2017). Evolution of influenza a virus by mutation and re-assortment. Int. J. Mol. Sci..

[10] Balgi A.D., Wang J., Cheng D.Y.H., Ma C., Pfeifer T.A., Shimizu Y., Anderson H.J., Pinto L.H., Lamb R.A., DeGrado W.F. (2013). Inhibitors of the Influenza A Virus M2 Proton Channel Discovered Using a High-Throughput Yeast Growth Restoration Assay. PLoS ONE.

[11] Hayden F.G. (2012). Newer influenza antivirals, biotherapeutics and combinations. Influenza Other Respir. Viruses.

[12] Masuda H., Suzuki H., Oshitani H., Saito R., Kawasaki S., Nishikawa M., Satoh H. (2000). Incidence of amantadine-resistant influenza A viruses in sentinel surveillance sites and nursing homes in Niigata, Japan. Microbiol. Immunol..

[13] Saito R., Sakai T., Sato I., Sano Y., Oshitani H., Sato M., Suzuki H. (2003). Frequency of amantadine-resistant influenza A viruses during two seasons featuring cocirculation of H1N1 and H3N2. J. Clin. Microbiol..

[14] Lee J., Young J.S., Jeung H.P., Lee J.H., Yun H.B., Song M.S., Oh T.K., Han H.S., Pascua P.N.Q., Choi Y.K. (2008). Emergence of amantadine-resistant H3N2 avian influenza A virus in South Korea. J. Clin. Microbiol..

[15] Zaraket H., Saito R., Suzuki Y., Baranovich T., Dapat C., Caperig-Dapat I., Suzuki H. (2010). Genetic makeup of amantadine-resistant and oseltamivir-resistant human influenza A/H1N1 viruses. J. Clin. Microbiol..

[16] Gultyaev A.P., Fouchier R.A.M., Olsthoorn R.C.L. (2010). Influenza virus RNA structure: Unique and common features. Int. Rev. Immunol..

[17] Ferhadian D., Contrant M., Printz-Schweigert A., Smyth R.P., Paillart J.C., Marquet R. (2018). Structural and functional motifs in influenza virus RNAs. Front. Microbiol..

[18] Jiang T., Nogales A., Baker S.F., Martinez-Sobrido L., Turner D.H. (2016). Mutations designed by ensemble defect to misfold conserved RNA structures of influenza A segments 7 and 8 affect splicing and attenuate viral replication in cell culture. PLoS ONE.

[19] Priore S.F., Kierzek E., Kierzek R., Baman J.R., Moss W.N., Dela-Moss L.I., Turner D.H. (2013). Secondary structure of a conserved domain in the intron of influenza A NS1 mRNA. PLoS ONE.

[20] Piasecka J., Jarmolowicz A., Kierzek E. (2020). Organization of the Influenza A Virus Genomic RNA in the Viral Replication Cycle—Structure, Interactions, and Implications for the Emergence of New Strains. Pathogens.

[21] Tomescu A.I., Robb N.C., Hengrung N., Fodor E., Kapanidis A.N. (2014). Single-molecule FRET reveals a corkscrew RNA structure for the polymerase-bound influenza virus promoter. Proc. Natl. Acad. Sci. USA.

[22] Coloma R., Arranz R., De La Rosa-Trevín J.M., Sorzano C.O.S., Munier S., Carlero D., Naffakh N., Ortín J., Martín-Benito J. (2020). Structural insights into influenza A virus ribonucleoproteins reveal a processive helical track as transcription mechanism. Nat. Microbiol..

[23] Pflug A., Guilligay D., Reich S., Cusack S. (2014). Structure of influenza A polymerase bound to the viral RNA promoter. Nature.

[24] Kim H.J., Fodor E., Brownlee G.G., Seong B.L. (1997). Mutational analysis of the RNA-fork model of the influenza A virus vRNA promoter in vivo. J. Gen. Virol..

[25] Le Sage V., Kanarek J.P., Snyder D.J., Cooper V.S., Lakdawala S.S., Lee N. (2020). Mapping of Influenza Virus RNA-RNA Interactions Reveals a Flexible Network. Cell Rep..

[26] Wandzik J.M., Kouba T., Karuppasamy M., Pflug A., Drncova P., Provaznik J., Azevedo N., Cusack S. (2020). A structure-based model for the complete transcription cycle of influenza polymerase. Cell.

[27] Noble E., Mathews D.H., Chen J.L., Turner D.H., Takimoto T., Kim B. (2011). Biophysical Analysis of Influenza A Virus RNA Promoter at Physiological Temperatures. J. Biol. Chem..

[28] Crescenzo-Chaigne B., Barbezange C., van der Werf S. (2013). The Panhandle Formed by Influenza A and C Virus NS Non-Coding Regions Determines NS Segment Expression. PLoS ONE.

[29] Dadonaite B., Gilbertson B., Knight M.L., Trifkovic S., Rockman S., Laederach A., Brown L.E., Fodor E., Bauer D.L.V. (2019). The structure of the influenza A virus genome. Nat. Microbiol..

[30] Lee M.-K., Kim H.-E., Park E.-B., Lee J., Kim K.-H., Lim K., Yum S., Lee Y.-H., Kang S.-J., Lee J.-H. (2016). Structural features of influenza A virus panhandle RNA enabling the activation of RIG-I independently of 5′-triphosphate. Nucleic Acids Res..

[31] Michalak P., Soszynska-Jozwiak M., Biala E., Moss W.N.W.N., Kesy J., Szutkowska B., Lenartowicz E., Kierzek R., Kierzek E. (2019). Secondary structure of the segment 5 genomic RNA of influenza A virus and its application for designing antisense oligonucleotides. Sci. Rep..

[32] Ruszkowska A., Lenartowicz E., Moss W.N., Kierzek R., Kierzek E. (2016). Secondary structure model of the naked segment 7 influenza A virus genomic RNA. Biochem. J..

[33] Lenartowicz E., Kesy J., Ruszkowska A., Soszynska-Jozwiak M., Michalak P., Moss W.N., Turner D.H., Kierzek R., Kierzek E. (2016). Self-folding of naked segment 8 genomic RNA of influenza a virus. PLoS ONE.

[34] Lenartowicz E., Nogales A., Kierzek E., Kierzek R., Martínez-Sobrido L., Turner D.H. (2016). Antisense Oligonucleotides Targeting Influenza A Segment 8 Genomic RNA Inhibit Viral Replication. Nucleic Acid Ther..

[35] Kierzek E. (2009). Binding of short oligonucleotides to RNA: Studies of the binding of common RNA structural motifs to isoenergetic microarrays. Biochemistry.

[36] Nakano M., Sugita Y., Kodera N., Miyamoto S., Muramoto Y., Wolf M., Noda T. (2021). Ultrastructure of influenza virus ribonucleoprotein complexes during viral RNA synthesis. Commun. Biol..

[37] Arranz R., Coloma R., Chichón F.J., Conesa J.J., Carrascosa J.L., Valpuesta J.M., Ortín J., Martín-Benito J. (2012). The structure of native influenza virion ribonucleoproteins. Science.

[38] Lee N., Le Sage V., Nanni A.V., Snyder D.J., Cooper V.S., Lakdawala S.S. (2017). Genome-wide analysis of influenza viral RNA and nucleoprotein association. Nucleic Acids Res..

[39] Le Sage V., Nanni A.V., Bhagwat A.R., Snyder D.J., Cooper V.S., Lakdawala S.S., Lee N. (2018). Non-uniform and non-random binding of nucleoprotein to influenza A and B viral RNA. Viruses.

[40] Moeller A., Kirchdoerfer R.N., Potter C.S., Carragher B., Wilson I.A. (2012). Organization of the influenza virus replication machinery. Science.

[41] Li X., Gu M., Zheng Q., Gao R., Liu X. (2021). Packaging signal of influenza A virus. Virol. J..

[42] Williams G.D., Townsend D., Wylie K.M., Kim P.J., Amarasinghe G.K., Kutluay S.B., Boon A.C.M. (2018). Nucleotide resolution mapping of influenza A virus nucleoprotein-RNA interactions reveals RNA features required for replication. Nat. Commun..

[43] Iselin L., Palmalux N., Kamel W., Simmonds P., Mohammed S., Castello A. (2022). Uncovering viral RNA–host cell interactions on a proteome-wide scale. Trends Biochem. Sci..

[44] Marc D. (2014). Influenza virus non-structural protein NS1: Interferon antagonism and beyond. J. Gen. Virol..

[45] Huang X., Zheng M., Wang P., Mok B.W.Y., Liu S., Lau S.Y., Chen P., Liu Y.C., Liu H., Chen Y. (2017). An NS-segment exonic splicing enhancer regulates influenza A virus replication in mammalian cells. Nat. Commun..

[46] Kuo R.L., Zhao C., Malur M., Krug R.M. (2010). Influenza IAV strains that circulate in humans differ in the ability of their NS1 proteins to block the activation of IRF3 and interferon-β transcription. Virology.

[47] Hale B.G., Randall R.E., Ortin J., Jackson D. (2008). The multifunctional NS1 protein of influenza A viruses. J. Gen. Virol..

[48] Lu Z.J., Gloor J.W., Mathews D.H. (2009). Improved RNA secondary structure prediction by maximizing expected pair accuracy. RNA.

[49] Hajiaghayi M., Condon A., Hoos H.H. (2012). Analysis of energy-based algorithms for RNA secondary structure prediction. BMC Bioinform..

[50] Soszynska-Jozwiak M., Pszczola M., Piasecka J., Peterson J.M., Moss W.N., Taras-Goslinska K., Kierzek R., Kierzek E. (2021). Universal and strain specific structure features of segment 8 genomic RNA of influenza A virus–application of 4-thiouridine photocrosslinking. J. Biol. Chem..

[51] Leamy K.A., Assmann S.M., Mathews D.H., Bevilacqua P.C. (2016). Bridging the gap between in vitro and in vivo RNA folding. Q. Rev. Biophys..

[52] Bae S.H., Cheong H.K., Lee J.H., Cheong C., Kainosho M., Choi B.S. (2001). Structural features of an influenza virus promoter and their implications for viral RNA synthesis. Proc. Natl. Acad. Sci. USA.

[53] Liu G., Zhou Y. (2019). Cytoplasm and Beyond: Dynamic Innate Immune Sensing of Influenza A Virus by RIG-I. J. Virol..

[54] Mathews D.H. (2014). RNA secondary structure analysis using RNAstructure. Curr. Protoc. Bioinform..

[55] Mathews D.H. (2014). Using the RNAstructure Software Package to Predict Conserved RNA Structures. Curr. Protoc. Bioinform..

[56] Hutchinson E.C., von Kirchbach J.C., Gog J.R., Digard P. (2010). Genome packaging in influenza A virus. J. Gen. Virol..

[57] Dias N., Stein C.A. (2002). Antisense oligonucleotides: Basic concepts and mechanisms. Mol. Cancer Ther..

[58] Hussain M., Galvin H.D., Haw T.Y., Nutsford A.N., Husain M. (2017). Drug resistance in influenza A virus: The epidemiology and management. Infect. Drug Resist..

[59] Tarn W.Y., Cheng Y., Ko S.H., Huang L.M. (2021). Antisense oligonucleotide-based therapy of viral infections. Pharmaceutics.

[60] Vickers T.A., Wyatt J.R., Freier S.M. (2000). Effects of RNA secondary structure on cellular antisense activity. Nucleic Acids Res..

[61] Lin X., Liu Y., Chemparathy A., Pande T., La Russa M., Qi L.S. (2021). A comprehensive analysis and resource to use CRISPR-Cas13 for broad-spectrum targeting of RNA viruses. Cell Rep. Med..

[62] Freije C.A., Myhrvold C., Boehm C.K., Lin A.E., Welch N.L., Carter A., Metsky H.C., Luo C.Y., Abudayyeh O.O., Gootenberg J.S. (2019). Programmable Inhibition and Detection of RNA Viruses Using Cas13. Mol. Cell.

[63] Abudayyeh O.O., Gootenberg J.S., Konermann S., Joung J., Slaymaker I.M., Cox D.B.T., Shmakov S., Makarova K.S., Semenova E., Minakhin L. (2016). C2c2 is a single-component programmable RNA-guided RNA-targeting CRISPR effector. Science.

[64] Abbott T.R., Dhamdhere G., Liu Y., Lin X., Goudy L., Zeng L., Chemparathy A., Chmura S., Heaton N.S., Debs R. (2020). Development of CRISPR as an Antiviral Strategy to Combat SARS-CoV-2 and Influenza. Cell.

[65] Wessels H.-H., Méndez-Mancilla A., Guo X., Legut M., Daniloski Z., Sanjana N.E. (2020). Massively parallel Cas13 screens reveal principles for guide RNA design. Nat. Biotechnol..

[66] Kesy J., Patil K.M.K.M., Kumar S.R., Shu Z., Yong H.Y.H.Y., Zimmermann L., Ong A.A.L.A.A.L., Toh D.-F.K.D.F.K., Krishna M.S.M.S., Yang L. (2019). A Short Chemically Modified dsRNA-Binding PNA (dbPNA) Inhibits Influenza Viral Replication by Targeting Viral RNA Panhandle Structure. Bioconjugate Chem..

[67] Meyer S.M., Williams C.C., Akahori Y., Tanaka T., Aikawa H., Tong Y., Childs-Disney J.L., Disney M.D. (2020). Small molecule recognition of disease-relevant RNA structures. Chem. Soc. Rev..

[68] Umuhire Juru A., Hargrove A.E. (2021). Frameworks for targeting RNA with small molecules. J. Biol. Chem..

[69] Warner K.D., Hajdin C.E., Weeks K.M. (2018). Principles for targeting RNA with drug-like small molecules. Nat. Rev. Drug Discov..

[70] Klimov A., Balish A., Veguilla V., Sun H., Schiffer J., Lu X., Kawaoka Y., Neumann G. (2012). Influenza Virus Titration, Antigenic Characterization, and Serological Methods for Antibody Detection. Influenza Virus: Methods and Protocols.

[71] Shatzkes K., Teferedegne B., Murata H. (2014). A simple, inexpensive method for preparing cell lysates suitable for downstream reverse transcription quantitative PCR. Sci. Rep..

[72] Kierzek E., Kierzek R. (2003). The synthesis of oligoribonucleotides containing N6-alkyladenosines and 2-methylthio-N6-alkyladenosines via post-synthetic modification of precursor oligomers. Nucleic Acids Res..

[73] Kierzek E., Kierzek R. (2003). The thermodynamic stability of RNA duplexes and hairpins containing N6-alkyladenosines and 2-methylthio-N6-alkyladenosines. Nucleic Acids Res..

[74] Fratczak A., Kierzek R., Kierzek E. (2009). LNA-modified primers drastically improve hybridization to target RNA and reverse transcription. Biochemistry.

[75] Soszynska-Jozwiak M., Michalak P., Moss W.N.W.N., Kierzek R., Kesy J., Kierzek E. (2017). Influenza virus segment 5 (+)RNA-Secondary structure and new targets for antiviral strategies. Sci. Rep..

[76] Beutner G.L., Kuethe J.T., Yasuda N. (2007). A practical method for preparation of 4-hydroxyquinolinone esters. J. Org. Chem..

[77] Kawakami E., Watanabe T., Fujii K., Goto H., Watanabe S., Noda T., Kawaoka Y. (2011). Strand-specific real-time RT-PCR for distinguishing influenza vRNA, cRNA, and mRNA. J. Virol. Methods.

[78] Vester D., Lagoda A., Hoffmann D., Seitz C., Heldt S., Bettenbrock K., Genzel Y., Reichl U. (2010). Real-time RT-qPCR assay for the analysis of human influenza A virus transcription and replication dynamics. J. Virol. Methods.

[79] Vasa S.M., Guex N., Wilkinson K.A., Weeks K.M., Giddings M.C. (2008). ShapeFinder: A software system for high-throughput quantitative analysis of nucleic acid reactivity information resolved by capillary electrophoresis. RNA.

[80] Low J.T., Weeks K.M. (2010). SHAPE-directed RNA secondary structure prediction. Methods.

[81] Reuter J.S., Mathews D.H. (2010). RNAstructure: Software for RNA secondary structure prediction and analysis. BMC Bioinformatics.

[82] Mathews D.H., Disney M.D., Childs J.L., Schroeder S.J., Zuker M., Turner D.H. (2004). Incorporating chemical modification constraints into a dynamic programming algorithm for prediction of RNA secondary structure. Proc. Natl. Acad. Sci. USA.

[83] Turner D.H., Mathews D.H. (2009). The nearest neighbor parameter database for predicting stability of nucleic acid secondary structure. Nucleic Acids Res..

[84] Byun Y., Han K. (2006). PseudoViewer: Web application and web service for visualizing RNA pseudoknots and secondary structures. Nucleic Acids Res..

[85] Robinson J.T., Thorvaldsdóttir H., Winckler W., Guttman M., Lander E.S., Getz G., Mesirov J.P. (2011). Integrative Genome Viewer. Nat. Biotechnol..

[86] Huynen M., Gutell R., Konings D. (1997). Assessing the reliability of RNA folding using statistical mechanics. J. Mol. Biol..

[87] Mathews D.H. (2004). Using an RNA secondary structure partition function to determine confidence in base pairs predicted by free energy minimization. RNA.

