# Supplementary material for: Secondary Structure of Influenza A Virus Genomic Segment 8 RNA Folded in a Cellular Environment
Source: Int J Mol Sci. 2022 Feb 23;23(5):2452. doi: 10.3390/ijms23052452 (PMC8910647; doi:10.3390/ijms23052452)
Supplement: Supplementary file 1 [file ijms-23-02452-s001.zip › Supplementary_Data/S1_Text.pdf]

Supporting information

Secondary structure of influenza A virus genomic segment 8 RNA folded in a cellular environment

Barbara Szutkowska[1], Klaudia Wieczorek[1], Ryszard Kierzek[1], Pawel Zmora[1], Jake M. Peterson[2], Walter N. Moss[2], David H. Mathews[3] and Elzbieta Kierzek[1*]

[1] Institute of Bioorganic Chemistry, Polish Academy of Sciences, Noskowskiego 12/14, 61-704 Poznan, Poland

[2] Roy J. Carver Department of Biophysics, Biochemistry and Molecular Biology, Iowa State University, Ames, IA 50011, United States of America

[3] Department of Biochemistry & Biophysics and Center for RNA Biology, 601 Elmwood Avenue, Box 712, School of Medicine and Dentistry, University of Rochester, Rochester, NY 14642

* Corresponding author

E-mail: elzbieta.kierzek@ibch.poznan.pl (EK)

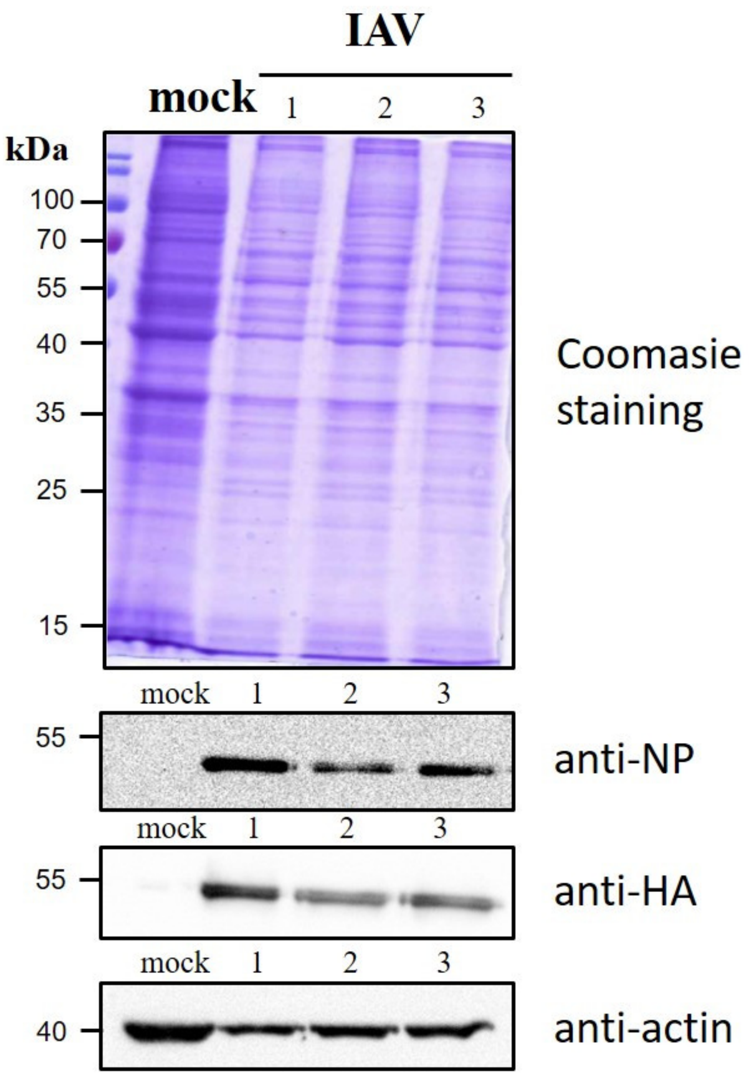

Figure S1. The IAV proteins expression in the infected cell lysates. The MDCK cells were infected with influenza virus A/California/04/2009 at MOI 0.01 and incubated for 24 hours. Next, the cells were treated with lysis buffer, and the total protein was stained with Coomasie blue (Bio-Rad). Additionally, the IAV proteins expression was detected by Western blot analysis using anti-NP (Thermo Scientific) and anti-HA (Merck) antibodies. Expression of actin, detected with anti-actin antibody (Thermo Scientific), served as a loading control.

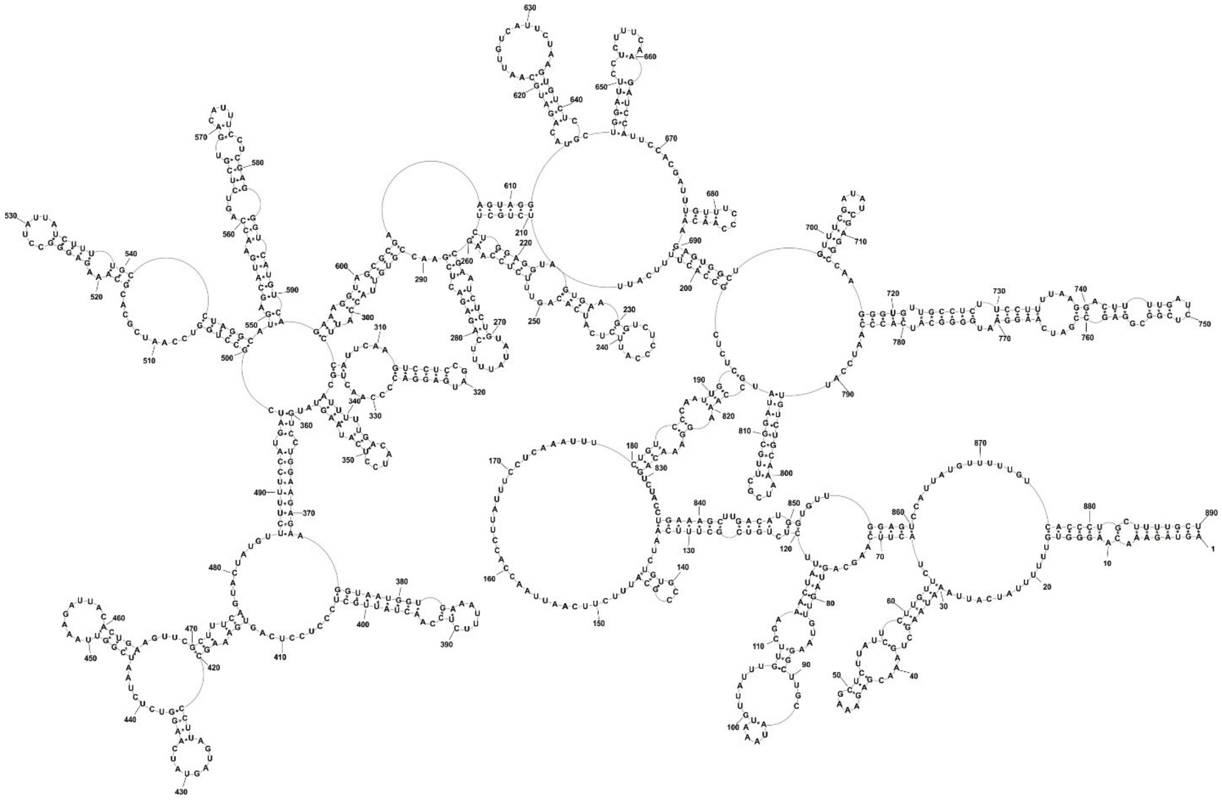

Figure S2. MEA global structure of vRNA8 A/California/04/2009 folded according to the experimental data. For the probability file calculation partition function in RNAStructure (6.2) experimental data (SHAPE and DMS), as well as panhandle base-pairing, were used. Visualization via PseudoViewer.

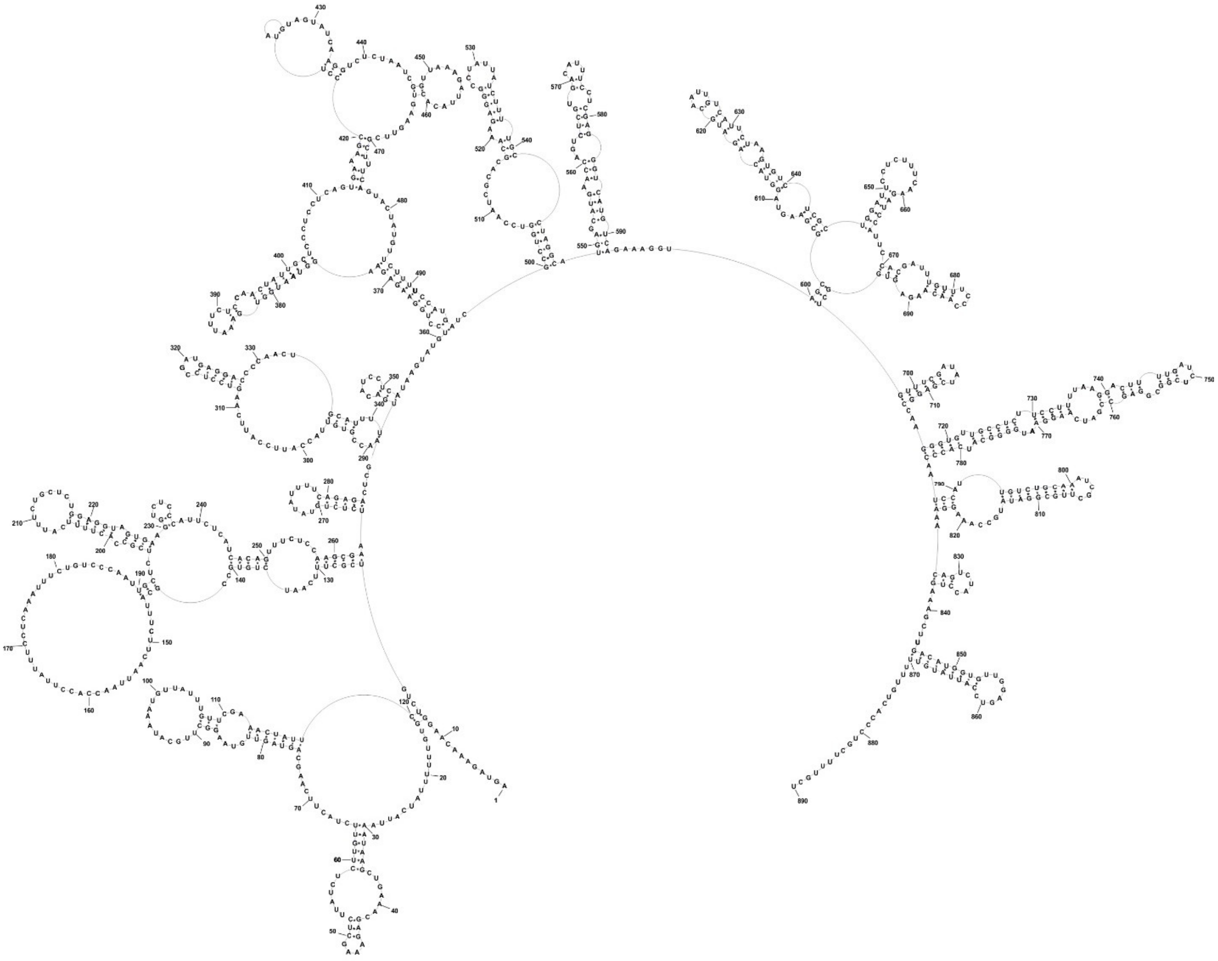

Figure S3. MEA local structure of vRNA8 A/California/04/2009 folded according to the experimental data. For the probability file calculation partition function in RNAStructure (6.2) experimental data (SHAPE and DMS) with maximum pairing distance set to 150 nt. The panhandle region was excluded from the calculation. Visualization via PseudoViewer.

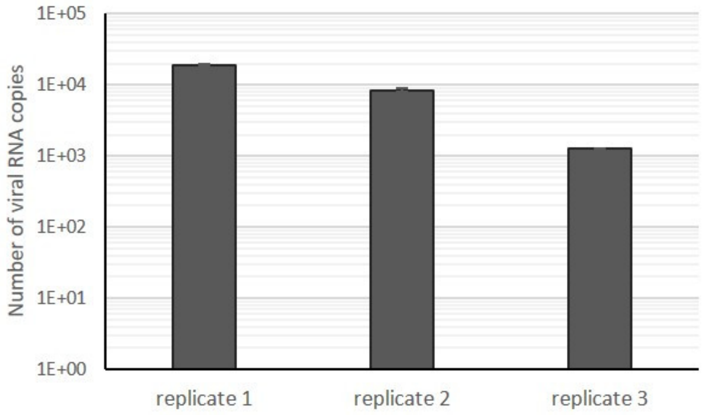

Figure S4. Amount of viral RNA8 copies in the independent replicates. The copies of vRNA8 were analyzed by RT-qPCR. The mean was calculated from three technical repeats by independent RT-qPCR reactions and the standard deviation is shown.
