# Supplementary figures and images for: Secondary Structure of Influenza A Virus Genomic Segment 8 RNA Folded in a Cellular Environment
Source: Int J Mol Sci. 2022 Feb 23;23(5):2452. doi: 10.3390/ijms23052452 (PMC8910647; doi:10.3390/ijms23052452)

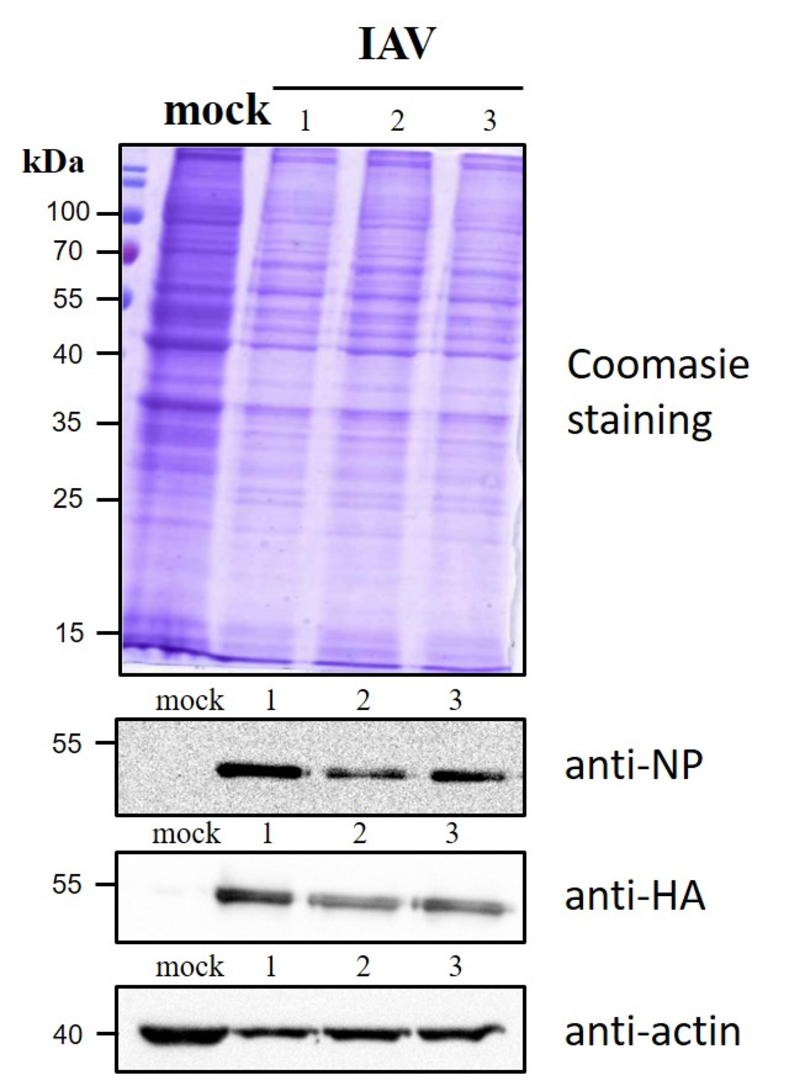

Supplement: Supplementary file 1 [file ijms-23-02452-s001.zip › Supplementary_Data/FigS1.tif.tif]

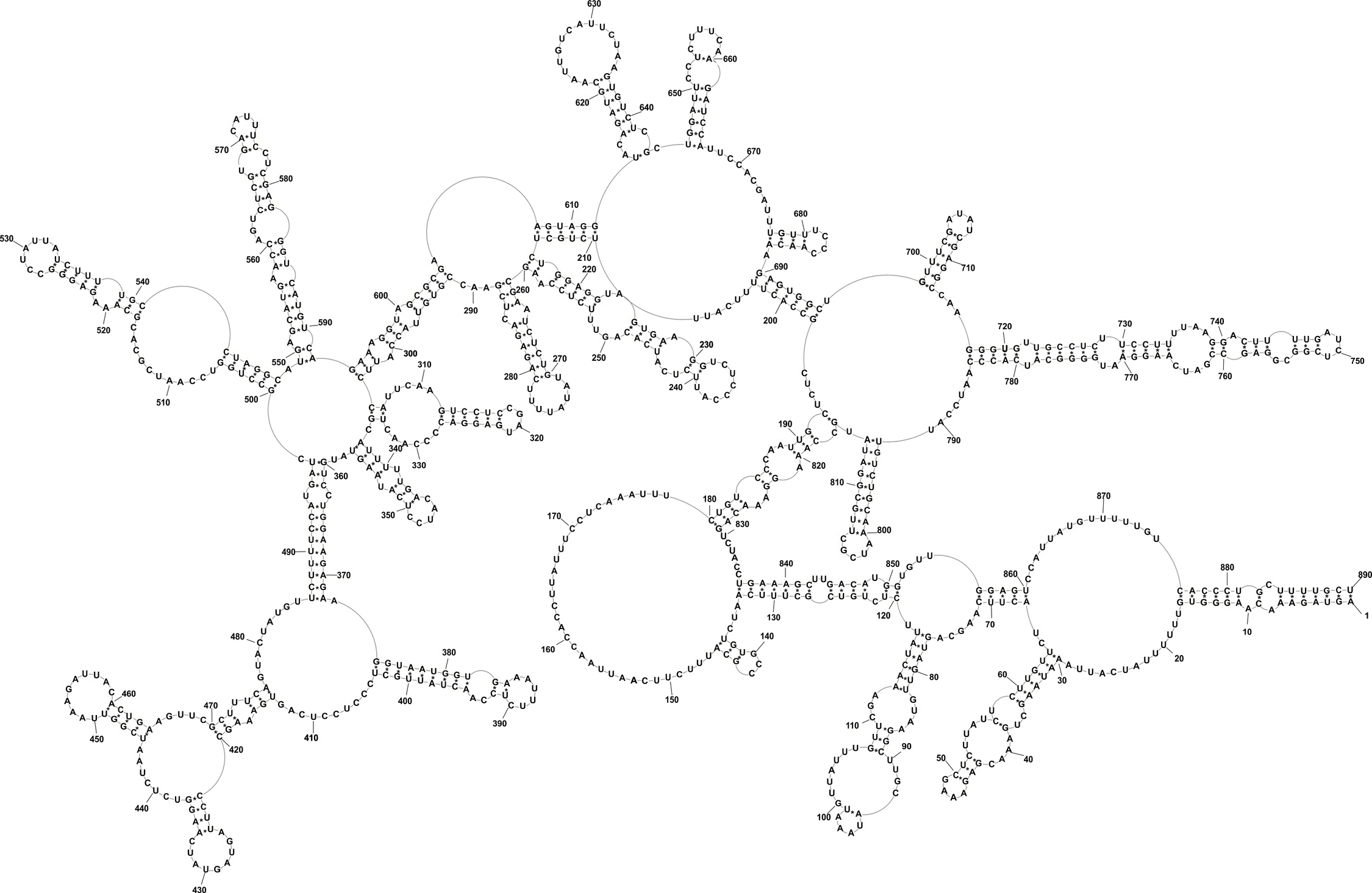

Supplement: Supplementary file 1 [file ijms-23-02452-s001.zip › Supplementary_Data/FigS2.tif.tif]

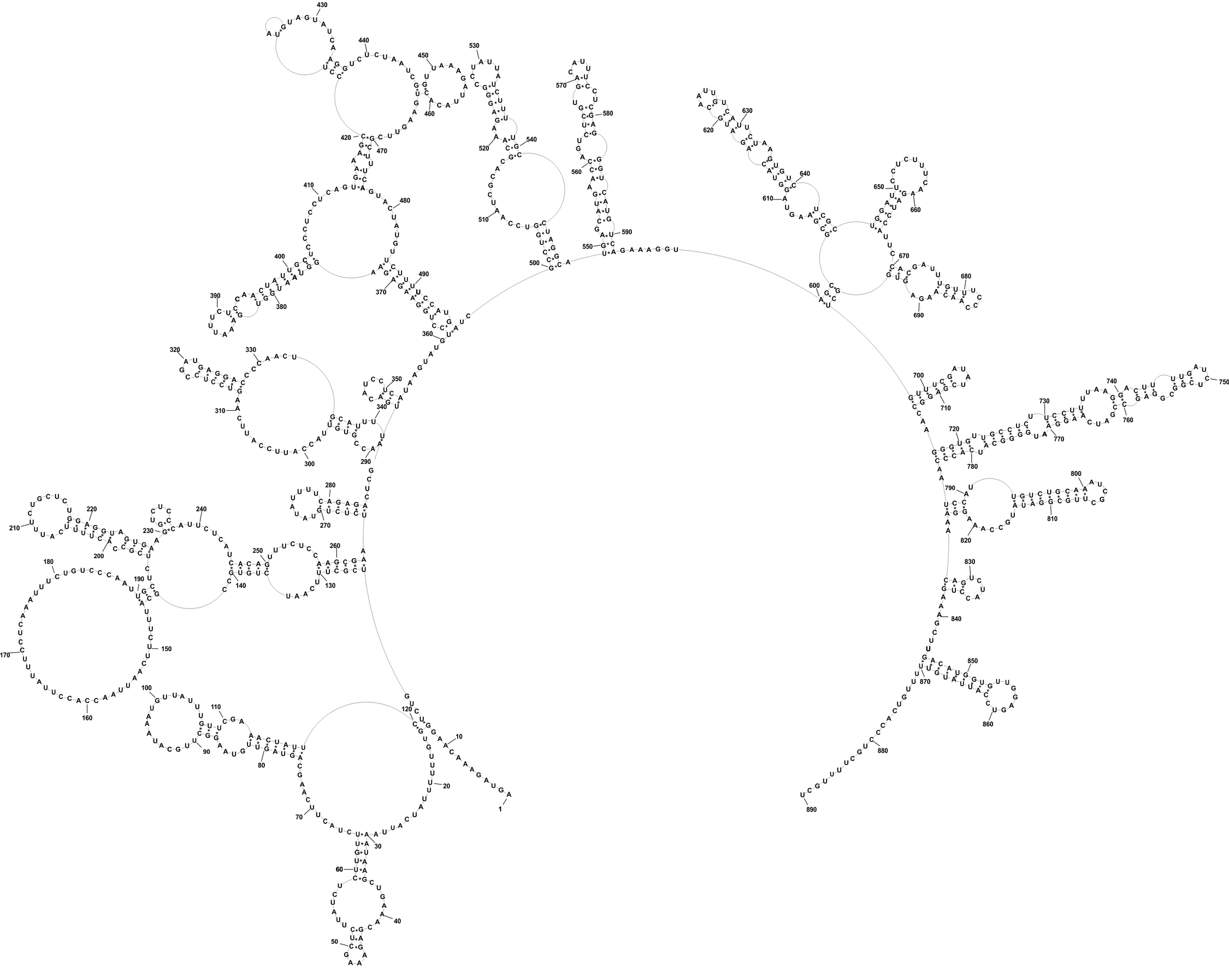

Supplement: Supplementary file 1 [file ijms-23-02452-s001.zip › Supplementary_Data/FigS3.tif.tif]

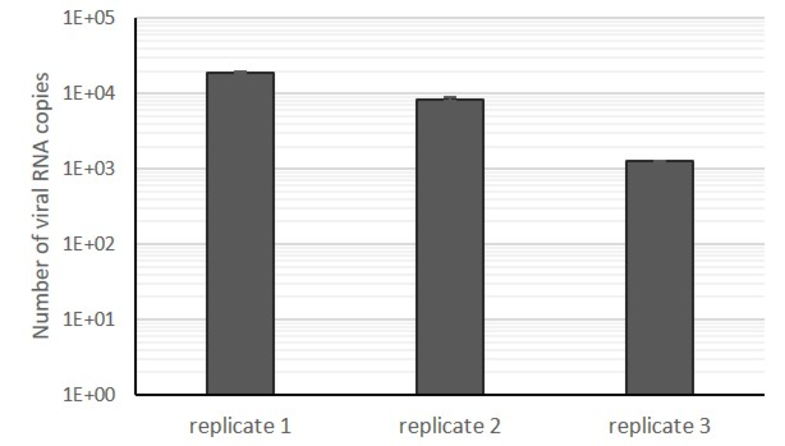

Supplement: Supplementary file 1 [file ijms-23-02452-s001.zip › Supplementary_Data/FigS4.tif.tif]
